# Nanomaterial-Enabled Modulation of Tumor-Associated Macrophages and Dendritic Cells to Enhance Cancer Immunotherapy

**DOI:** 10.3390/nano16030172

**Published:** 2026-01-27

**Authors:** Anbu Mozhi Thamizhchelvan, Kory Wells, Jacob Pham, Ashan Galhena, Woojin Kim

**Affiliations:** 1 Department of Radiology and Imaging Sciences, School of Medicine, Emory University, Atlanta, GA 30322, USA; jqpham5@emory.edu (J.P.); ashan.galhena@emory.edu (A.G.); woojin.kim@emory.edu (W.K.); 2 Department of Surgery, School of Medicine, Emory University, Atlanta, GA 30322, USA; kory.wells@emory.edu

**Keywords:** tumor microenvironment, tumor-associated macrophages, dendritic cells, M2-like phenotype, nanoparticle, T-cell priming

## Abstract

Tumor-associated macrophages (TAMs) and dendritic cells (DCs) play pivotal roles in shaping the tumor immune microenvironment, often contributing to immunosuppression and therapy resistance. Recent advances in nanotechnology have enabled precise modulation of these immune populations, offering a promising avenue to enhance the efficacy of cancer immunotherapy. Nano-enabled platforms can reprogram TAMs from a pro-tumorigenic M2-like phenotype to an anti-tumorigenic M1-like state, thereby restoring their capacity to phagocytose tumor cells and produce pro-inflammatory cytokines. Concurrently, nanomaterials can enhance DC activation and antigen presentation, promoting robust T-cell priming and adaptive immune responses. Various nanocarriers, including liposomes, polymeric nanoparticles, and inorganic nanostructures, have been engineered to deliver immune modulators, nucleic acids, or tumor antigens selectively to TAMs and DCs within the tumor microenvironment. These strategies have demonstrated synergistic effects when combined with immune checkpoint blockade or cytokine therapy, resulting in improved tumor regression and long-term immunological memory in preclinical models. Despite these promising outcomes, challenges remain regarding nanomaterial biocompatibility, targeted delivery efficiency, and potential off-target immune activation. Ongoing research is focused on optimizing nanoparticle physicochemical properties, surface functionalization, and multi-modal delivery systems to overcome these limitations. This review highlights recent advances in nano-enabled modulation of TAMs and DCs, emphasizing mechanistic insights, therapeutic outcomes, and translational potential. By integrating nanotechnology with immunotherapy, these approaches offer a powerful strategy to overcome tumor immune evasion, paving the way for more effective and personalized cancer treatments.

## 1. Introduction

Cancer immunotherapy has reshaped contemporary oncology by harnessing the body’s innate immune mechanisms to recognize and eliminate malignant cells. Immune checkpoint inhibitors (ICIs), adoptive cell transfer, dendritic cell (DC) vaccines, oncolytic viruses, and cytokine-based therapies have generated unprecedented responses across various cancers, including melanoma, lung cancer, renal carcinoma, and hematologic malignancies [1,2,3,4,5]. Yet, despite transformative success in selected patient populations, most individuals do not experience a durable benefit. A principal barrier to universal efficacy of immunotherapy is the immunosuppressive tumor microenvironment (TME), which actively impairs immune activation, suppresses antigen presentation, and restricts effector T-cell infiltration [6,7,8]. Addressing these suppressive mechanisms is therefore essential for unlocking the full therapeutic potential of immunotherapy.

The TME is a dynamic and highly heterogeneous ecosystem, composed of not only malignant cells, but also stromal elements, extracellular matrix components, vasculature, and a diverse array of immune populations [9]. Within this milieu, tumor-associated macrophages (TAMs) and dendritic cells occupy central regulatory positions and profoundly influence tumor immunity through their functional plasticity, antigen-presentation capacity, and crosstalk with T cells [10,11,12,13]. TAMs often represent the predominant immune population in solid tumors, comprising up to ~50% of infiltrating leukocytes, and accumulate under the influence of tumor-derived chemokines such as CCL2 and CSF-1, as well as stromal cues [14,15]. These macrophages are commonly skewed toward an M2-like, immunoregulatory phenotype characterized by suppression of effector T-cell function, promotion of angiogenesis, extracellular matrix remodeling, and facilitation of metastasis [16,17]. In contrast, classically activated M1-like macrophages support antitumor immunity via nitric oxide production, secretion of inflammatory cytokines, and robust antigen presentation [17]. Importantly, enrichment of M2-like TAMs is frequently associated with poor clinical prognosis, resistance to systemic therapy, and reduced responsiveness to immune checkpoint inhibitors, underscoring their value as a therapeutic target [15,18].

DCs, arguably the most potent antigen-presenting cells, are similarly suppressed within the TME [19,20]. Tumors inhibit DC differentiation and maturation by downregulating co-stimulatory signals, impairing cross-presentation pathways, and reducing expression of major histocompatibility complex (MHC) molecules [21,22]. These dysfunctional or tolerogenic DC subsets exhibit attenuated ability to prime cytotoxic T lymphocytes (CTLs), enabling tumors to evade immune surveillance [23]. Together, TAMs and DCs form an interconnected immunoregulatory axis whose dysfunction establishes an environment conducive to immune escape. Multiple immunosuppressive cues within the TME reinforce this dysfunctional state. Soluble mediators such as IL-10, transforming growth factor-β (TGF-β), and vascular endothelial growth factor (VEGF) suppress DC activation and promote M2-like macrophage polarization [24,25]. Metabolic stressors, including lactate, adenosine, and kynurenine, further inhibit T-cell effector functions while reinforcing TAM-mediated tolerance [26]. Additionally, structural barriers, such as hypoxia, disordered vasculature, and a stiff or fibrotic extracellular matrix, restrict immune and drug penetration [27,28]. Collectively, these features create a multilayered network of suppression that next-generation immunotherapies must overcome.

Nanotechnology has emerged as a powerful and versatile platform capable of addressing these barriers. Nanomaterials, including liposomes, polymeric nanoparticles, dendrimers, inorganic nanostructures, and hybrid nano–bio constructs, offer tunable physicochemical properties that enable targeted delivery, controlled release, and immune-cell-specific modulation [29,30,31,32,33,34,35,36,37,38,39]. Their capacity to co-deliver immune agonists, antigens, nucleic acids, or cytokines directly to TAMs or DCs makes nanotechnology an ideal strategy for simultaneously reprogramming multiple components of the TME [40,41,42,43,44].

In TAMs, nanoparticles have demonstrated strong potential to re-educate macrophages from an M2-like to an M1-like phenotype. Platforms delivering toll-like receptor (TLR) agonists, CSF-1R inhibitors, PI3Kγ antagonists, STING agonists, or cytokine-encoding RNA constructs have shown robust induction of pro-inflammatory programs and improved antigen presentation [45,46,47,48,49]. Such reprogramming not only suppresses tumor progression but also enhances T-cell infiltration and potentiates ICI efficacy across diverse tumor models. Similarly, nanomaterials designed to target DCs have improved tumor antigen uptake, enhanced cross-presentation efficiency, and strengthened CD8^+^ T-cell responses compared with conventional vaccine formulations [50].

Beyond direct modulation of macrophages and DCs, nanotechnology offers broader opportunities to remodel the TME. Hypoxia-responsive nanocarriers can deliver oxygen, generate reactive oxygen species, or promote vascular normalization to restore immune functionality [51,52,53], while matrix-degrading nanostructures facilitate deeper immune and drug infiltration [54]. Multifunctional nanoparticles capable of co-delivering chemotherapeutics, ICIs, antigens, and immune agonists enable simultaneous disruption of tumor cells and immunosuppressive circuits [55]. Moreover, stimuli-responsive nanomaterials activated by pH, redox gradients, enzymes, light, or ultrasound enable spatially and temporally precise release of therapeutic payloads, improving efficacy while limiting systemic toxicity [56].

The rationale for nano-enabled modulation of TAMs and DCs arises from the unique ability of nanomaterials to integrate targeted delivery, immune activation, and microenvironmental remodeling. By promoting DC maturation and antigen presentation, repolarizing TAMs toward pro-inflammatory phenotypes, and enhancing T-cell infiltration, nanotechnology can convert immunologically “cold” tumors into “hot,” immune-responsive tumors characterized by robust effector cell infiltration and the production of inflammatory cytokines [57,58,59,60,61]. These transformations have been shown to sensitize tumors to ICIs, adoptive cell therapy, and therapeutic cancer vaccines [50,62,63,64,65,66,67]. Despite these advances, key translational barriers remain, including optimizing nanoparticle biodistribution, ensuring long-term safety and biocompatibility, and establishing scalable Good Manufacturing Practice (GMP) compliant production. Ongoing innovations in biomimetic surface engineering, ligand-directed targeting, and next-generation hybrid materials are poised to improve clinical translation and therapeutic impact [68,69]. Ultimately, the convergence of nanotechnology with immunology offers a versatile and powerful platform for overcoming TME-mediated resistance and unlocking the full potential of cancer immunotherapy.

In this review, we provide a comprehensive and critical overview of nano-enabled strategies for modulating TAMs and DCs to enhance cancer immunotherapy. We first examine the functional roles of TAMs and DCs in orchestrating TME immunosuppression, then discuss current classes of nanomaterials and their mechanisms for reprogramming these cells. We highlight emerging nano-immunotherapy platforms, synergistic combinations with ICIs and vaccines, and recent advances in stimuli-responsive and biomimetic systems. Finally, we address translational challenges, safety considerations, and future directions for integrating nanotechnology with next-generation immune-based therapies.

## 2. Tumor Immune Microenvironment (TIME)

A detailed understanding of the tumor immune microenvironment (TIME) is essential for deciphering how tumors escape immunosurveillance and for designing therapies that counteract these mechanisms. The TIME comprises malignant cells, stromal components, vasculature, and diverse immune populations whose coordinated or antagonistic interactions shape clinical outcomes [70,71]. Within this network, tumor-associated macrophages (TAMs) and dendritic cells (DCs) are particularly influential because their functional states determine whether the microenvironment supports immune activation or immune suppression.

### 2.1. Tumor-Associated Macrophages (TAMs): Origin, Polarization, and Function

TAMs are among the most abundant immune cells in solid tumors, often representing 30–50% of infiltrating leukocytes [70,71]. They arise from resident tissue macrophages and circulating monocytes recruited by chemokines such as CCL2 and growth factors, including CSF-1 [71,72]. Once within the tumor, macrophages undergo extensive reprogramming driven by cytokines, metabolites, hypoxia, and stromal cues. Their functional diversity is commonly described along an M1-like to M2-like spectrum. M1-like TAMs, induced by IFN-γ or microbial components such as lipopolysaccharide (LPS), produce interleukin-12 (IL-12) and tumor necrosis factor-α (TNF-α), increase nitric oxide (NO) generation, and support antitumor T-cell responses [70]. M2-like TAMs, stimulated by IL-4, IL-10, or tumor-derived lactate, promote angiogenesis (via VEGF, PDGF), extracellular matrix remodeling, and immunosuppression through pathways involving arginase-1 (Arg1), indoleamine 2,3-dioxygenase (IDO), and programmed death-ligand 1 (PD-L1) [70,72,73]. They also recruit regulatory T cells via CCL17 and CCL22, reinforcing local tolerance.

Importantly, TAMs exhibit high plasticity and can transition between phenotypes in response to evolving environmental cues. This plasticity underlies major therapeutic strategies aimed at preventing monocyte recruitment, depleting macrophages, or, most promisingly, reprogramming M2-like TAMs into pro-inflammatory, antigen-presenting M1-like states to enhance responsiveness to immune checkpoint inhibitors (ICIs) [71,72].

### 2.2. Dendritic Cells (DCs): Function, Impairment, and Therapeutic Potential

DCs serve as the primary antigen-presenting cells, priming naïve T cells and coordinating effector and memory responses [12,74]. Conventional type 1 DCs (cDC1s) excel in cross-presentation to CD8^+^ T cells, whereas cDC2s preferentially activate CD4^+^ helper T cells [74]. Within tumors, DCs frequently remain immature or tolerogenic due to tumor-secreted immunosuppressive mediators such as VEGF, IL-10, and TGF-β [75]. Suppressed co-stimulatory signaling, impaired MHC expression, and metabolic stressors limit their capacity to prime cytotoxic T lymphocytes, enabling tumors to escape immune detection. This dysfunction is particularly pronounced in pancreatic cancers and hematologic malignancies [74,75]. To overcome these barriers, emerging therapeutic strategies aim to restore DC differentiation and activation. Lipid nanoparticle-based vaccines and mRNA platforms promote DC maturation, enhance antigen presentation, and stimulate robust T-cell responses within the TIME [49].

### 2.3. Integrated Roles of TAMs and DCs in Immune Evasion and Therapy

TAMs and DCs operate within a tightly coordinated regulatory axis that fundamentally shapes immune outcomes in tumors. Tumors exploit this axis by simultaneously inhibiting DC maturation and driving TAMs toward immunosuppressive phenotypes, thereby blocking T-cell priming, restricting T-cell infiltration, and reinforcing tolerance [76,77]. Such mechanisms contribute to immune evasion in multiple cancers, including bladder and ovarian malignancies, where disrupted antigen trafficking and stromal barriers severely limit adaptive immunity [76,78]. Modern immunotherapy increasingly focuses on dual modulation of TAMs and DCs, reflecting recognition that targeting a single immune cell type is often insufficient. Combined approaches such as TAM reprogramming coupled with DC activation have shown promise in shifting tumors from antigen-poor, immunosuppressed states (“cold” tumors) toward antigen-rich, T-cell-inflamed phenotypes (“hot” tumors) that respond more effectively to ICIs and other immune-based treatments [49,57,79].

## 3. Nanomaterial Platforms for Immunomodulation: Design Principles and Functional Mechanisms

Nanomaterials are emerging as versatile immunomodulatory platforms, capable of precisely reprogramming immune responses for vast therapeutic benefits. A diverse array of nanocarriers, including lipid, inorganic, organic, and hybrid systems, are engineered to deliver immunomodulatory cargoes. The efficacy of these platforms hinges on their rational design, where biophysical design parameters such as size, charge, shape, and surface chemistry are carefully tuned. These intrinsic properties dictate the nanoparticles’ biological fate in vivo, including circulation time, tumor accumulation, cellular uptake, and intracellular trafficking, thereby dictating their specific interaction with immune cells. This enables advanced targeting strategies aimed at key populations within the TME, such as enhancing antigen presentation in DCs for vaccination or reprogramming the immunosuppressive phenotype of TAMs.

Intrinsic nanoplatform properties are a significant determinant of TAM targeting because they govern both tumor accumulation via the Enhanced Permeation and Retention (EPR) effect and subsequent uptake by macrophages. Nanoparticles within a therapeutic size range (~20–200 nm) can passively extravasate through the leaky and irregular tumor vasculature and accumulate in tumor tissues due to the EPR effect, which arises from disordered endothelial gaps and poor lymphatic drainage in solid tumors [80,81,82,83]. This passive tumor accumulation is strongly influenced by nanoplatform properties such as size, surface charge, and circulation half-life, all of which affect extravasation and retention in the tumor interstitium [84,85]. Once within the tumor microenvironment, TAMs, which are abundant in many solid tumors, can engulf nanoparticles far more efficiently than cancer cells, effectively acting as cellular depots for nanotherapeutics and nanocarriers [86]. For example, polymeric-based nanoparticles accumulated in tumors via the EPR effect exhibited significantly higher nanoparticle uptake in TAM-rich tumors compared with TAM-deficient models, indicating that macrophage content determines intratumoral NP retention [87]. Similarly, systemically administered nanoliposomes carrying C6-ceramide (LipC6) were shown to modulate TAM phenotype and enhance anti-tumor immune responses in liver tumor models without the use of any targeting ligands. The uptake by TAMs was mediated entirely by the physicochemical properties and passive biodistribution of the nanoparticles [88]. Clinically approved superparamagnetic iron oxide nanoparticles such as ferumoxytol are also passively delivered to tumor sites via EPR and preferentially phagocytosed by TAMs, as evidenced by MRI signal changes correlating with macrophage infiltration in both preclinical models and patients [89,90]. Quantitative [Fe]MRI studies further confirmed that ferumoxytol SPIONs are readily internalized by F4/80high^+^CD11bhigh^+^ TAMs and exhibit peak tumor iron concentration at ~42 h post–intravenous administration, with negligible uptake in non-tumor tissues [90,91]. In addition, nanoparticle physicochemical features such as hydrophobicity, shape, and surface chemistry can modulate macrophage recognition and internalization, leading to enhanced phagocytic uptake independent of active targeting ligands [92,93,94,95]. Taken together, these findings demonstrate that nanoplatform properties per se governing EPR-mediated tumor accumulation and innate macrophage uptake are advantageous for targeting TAMs and provide a mechanistic rationale for designing immunomodulatory nanomedicines that exploit these intrinsic characteristics, rather than relying solely on active targeting moieties.

Lipid nanoparticles (LNPs) can function as powerful immunomodulatory platforms by transforming an inherent challenge into a distinct therapeutic advantage. Nano drug delivery systems (NDDS) are known to be easily captured by macrophages upon administration [96], a characteristic this strategy leverages to specifically target macrophages, including TAMs, for in situ genetic reprogramming. The rational design of these LNPs is key to their function. For instance, LNP/CAR Trop2 particles were engineered with specific biophysical parameters, including a spherical shape, a size of approximately 200 nm, and a near-neutral charge, which confers good serum stability [96]. The nanoparticle’s composition is also critical, incorporating cationic lipids and nuclear localization peptides (NLS) to ensure the plasmid DNA payload is successfully encapsulated and transfected into the target cells. This LNP-mediated strategy effectively reprograms TAMs, converting them into a pro-inflammatory M1 phenotype. This in situ programming method successfully converted most macrophages into an M1-type CAR-M phenotype, which in turn demonstrated specific phagocytic activity against cancer cells [96]. In addition to lipids, polymeric nanomaterials (PPNPs) have been engineered as an integrated immune-modulatory system, combining gene silencing with photodynamic therapy (PDT). One such nanoparticle is spherical with a hydrodynamic size of approximately 72.18 nm, a dimension that facilitates efficient cellular uptake and deep penetration into tumor tissues. The polymer is synthesized with a strong positive charge to electrostatically adsorb the negatively charged siRNA payload, but the final complex is formulated to have a neutral zeta potential, which is favorable for systemic circulation [97]. Furthermore, its chemical design incorporates a glutathione (GSH)-cleavable linker, making the nanoparticle responsive to the specific tumor microenvironment and enabling the accelerated, on-demand release of its therapeutic cargo. Regarding immune cell modulation, the platform’s strategy focuses on indirectly acting on dendritic cells (DCs). The PPNP serves as a photosensitizer that, upon activation by NIR-II laser irradiation, generates reactive oxygen species (ROS) to induce immunogenic cell death (ICD) in tumor cells. This ICD process releases danger-associated molecular patterns (DAMPs), which are then recognized by DCs. This recognition promotes DC maturation and the subsequent activation of T cells, effectively transforming the “cold” tumor microenvironment into a “hot,” immune-activated state [97].

Inorganic nanoparticles can also be utilized as immune-modulatory platforms, employing distinct biophysical designs and targeting strategies. For example, theragnostic gadolinium-based (AGuIX) nanoparticles are hybrid particles engineered for both diagnosis and therapy. They are extremely small, with a hydrodynamic diameter of approximately 5 nm. Their surface chemistry consists of an inorganic polysiloxane network (a “ceramic” base) grafted with around 15 organic gadolinium chelates. Specifically, DOTAGA chelates are used, which provide a high complexation constant comparable to clinical MRI contrast agents [98]. This structure has a near-neutral isoelectric point close to 7 and is designed to be biodegradable, as the Si-O-Si bonds hydrolyze at low concentrations, facilitating rapid renal clearance. This “theragnostic” action stems from the gadolinium: its paramagnetic properties make it an excellent T1-weighted MRI contrast agent for noninvasive tracking and tumor delineation, while its high-Z (high atomic number) nature acts as a potent radio-enhancer. When irradiated, it generates secondary radiation, focally amplifying the radiation dose within the tumor. This platform’s targeting strategy is passive; it is administered intravenously and relies on the EPR effect to accumulate in tumors. This IV route is a key advantage over platforms requiring direct intratumoral injection. The platform does not actively target immune cells like macrophages or dendritic cells. Instead, its immune-modulatory action is a secondary consequence of its radio-sensitizing effect, which preclinical data suggest induces immunogenic cell death and subsequently activates a systemic T-cell response [98]. In contrast, a different hybrid approach uses spherical calcium carbonate CaCO_3_ nanoparticles to directly activate T cells. These particles are significantly larger, with an inorganic core size of 110 nm +/− 20 pm and a final hydrodynamic size of approximately 200 nm. Their surface chemistry is a multi-layered hybrid design. The CaCO_3_ core is first coated with oleic acid, creating a hydrophobic interlayer, and then with a DSPE-PEG lipid coating. This design is critical, as it provides colloidal stability and slows the nanoparticle’s degradation within the acidic endosome. This ensures a sustained release of calcium, which is essential for T-cell activation rather than a transient surge. This lipid layer is also used to load PMA (12-myristate 13-acetate), a protein kinase C activator. The particle’s DSPE-PEG coating results in a negative surface charge, and its targeting strategy is active, not passive. Anti-PD-1 antibodies are conjugated to the surface to selectively target PD-1-positive T cells, such as those that are exhausted or tumor-infiltrating. The nanoparticles are internalized via PD-1 receptor-mediated, dynamin-dependent endocytosis. Once inside, the degrading core releases calcium and PMA, which co-activate the NFAT and NF-kB signaling pathways, respectively, mimicking natural T-cell activation signals. Studies within the tumor microenvironment confirmed that these nanoparticles were preferentially taken up by T cells compared to phagocytes, including macrophages and dendritic cells [99].

## 4. Nano-Enabled Reprogramming of TAMS

Reprogramming tumor-associated macrophages (TAMs) toward a pro-inflammatory, tumoricidal state is central to next-generation cancer immunotherapy [100,101]. Conventional approaches employing soluble small molecules, cytokines, or nucleic acids, such as signal transducer and activator of transcription 3 (STAT3) inhibitors, PI3Kγ antagonists, TLR agonists, CSF-1R inhibitors, and microRNAs (miR-155, miR-21, miR-146b) have shown promise in modulating intracellular signaling and transcriptional programs [102,103,104,105,106]. However, their clinical translation has been hampered by poor pharmacokinetics, rapid degradation, systemic toxicity, and insufficient accumulation within the tumor microenvironment (TME).

Nanotechnology offers versatile platforms that overcome these limitations by enabling targeted delivery, controlled release, and intracellular programming of TAMs. Lipid-based, polymeric, and hybrid nanoparticles have demonstrated efficient delivery of siRNA and mRNA to reprogram macrophages toward antitumor phenotypes through activation of NF-κB and JAK/STAT signaling [107,108,109,110]. In parallel, hybrid and functional nanomaterials have been shown to modulate additional pathways, including Notch signaling and IRF5-dependent transcription, enabling durable pro-inflammatory phenotypes [111,112,113]. Moreover, the intrinsic properties of certain nanomaterials, including metal oxides, carbon-based nanostructures, and biomimetic carriers, can directly modulate macrophage signaling via redox activity, lysosomal stress, or surface receptor interactions, independent of encapsulated cargo [113,114,115,116].

Functionally, TAM-reprogramming nanomaterials can be classified into three overlapping categories:

- Drug-mediated classical pathway engagement
- Material-Intrinsic Signaling
- Combinatorial multi-pathway platforms

The following sections outline these strategies, focusing on mechanistic pathways, representative nanoplatforms, and functional outcomes on TAMs and tumor immunity.

### 4.1. Drug-Mediated Classical Pathway Engagement

Classical pathway approaches use nanocarriers to deliver molecules such as STAT3/JAK-STAT, PI3Kγ, TLRs (agonists), CSF-1R inhibitors, and Notch modulators that directly modulate well-mapped signaling cascades in macrophages. These strategies aim for predictable transcriptional rewiring (cytokine/chemokine changes, antigen-presentation machinery, costimulatory molecule upregulation) by perturbing nodes that determine macrophage phenotype.

#### 4.1.1. Pi3kγ Inhibitor Delivery Systems

Among emerging strategies for TAM reprogramming, targeted inhibition of phosphoinositide-3-kinase gamma (PI3Kγ) has garnered considerable attention due to its central role in regulating macrophage-mediated immunosuppression. Small-molecule inhibitors, particularly IPI-549, have shown promise in restoring pro-inflammatory signaling in TAMs, yet their clinical translation is hindered by poor solubility, rapid clearance, and systemic toxicity. Nanomaterial-based delivery platforms provide a versatile solution by enhancing tumor accumulation, facilitating macrophage-specific uptake, and enabling controlled intracellular release. A notable example is an albumin-based nanoparticle “Nano-PI”, which co-encapsulates the PI3Kγ inhibitor IPI-549 and the chemotherapeutic paclitaxel. When administered along with α-PD1 therapy in dual breast cancer murine models, Nano-PI successfully restructured the immune landscape within both lymphoid tissues and primary tumors. This therapeutic approach produced sustained tumor regression and completely eradicated pulmonary metastatic lesions in the breast cancer mouse models. The PTX with IPI-549 combination stabilized the nanoparticle while promoting M2-to-M1 macrophage repolarization. Nano-PI enhanced drug delivery to lymph nodes and tumors, with preferential macrophage accumulation. Comprehensive immunophenotyping analysis demonstrated that Nano-PI combined with α-PD1 checkpoint blockade orchestrated significant immune microenvironment alterations, including macrophage phenotype switching from M2 to M1, expansion of both CD4^+^ and CD8^+^ T lymphocyte populations, increased B cell and dendritic cell infiltration, reduced regulatory T cell frequencies, and prevention of T cell exhaustion markers [47].

In another study, Jiang et al. [117] developed a dual-drug nanoformulation strategy to restructure the breast cancer microenvironment. They co-encapsulated silibinin, an anti-fibrotic compound targeting cancer-associated fibroblasts with immunomodulatory properties, and IPI-549 within AEAA-PEG-PCL nanocarriers for systemic delivery. In 4T1 tumor-bearing mice, this combination exhibited enhanced therapeutic efficacy compared to single-agent treatments, promoting tumor cell apoptosis while significantly reducing regulatory T cells and myeloid-derived suppressor cells. Additionally, the treatment normalized the tumor tissue architecture by suppressing angiogenesis, reducing fibrosis, and inhibiting collagen accumulation, collectively amplifying anti-tumor responses and representing a viable therapeutic strategy for breast cancer. Wang et al. [118] engineered an innovative core-shell nanosystem to overcome the challenge of co-delivering hydrophobic and hydrophilic immunomodulators. The developed nano-core has IPI549, a hydrophobic inhibitor, encapsulated by a hydrophilic TLR9 agonist, CpG-loaded metal-organic framework (MOF) shell. This biocompatible platform ensures coordinated intracellular delivery into TAMs, where the synergistic action of the payloads reprograms TAMs from M2 to M1 phenotype. These re-educated macrophages then orchestrate a comprehensive immune response, directly engaging innate immunity through phagocytosis and cytokine secretion while initiating adaptive immunity by presenting tumor antigens to recruit and activate cytotoxic T lymphocytes. Consequently, this nanosystem, combined with αPD-L1 therapy, triggers a potent anti-tumor response that effectively inhibits tumor growth and metastasis.

In a targeted approach, Li et al. [119] developed mannose-decorated porous hollow iron oxide nanoparticles (PHNPs) to deliver the PI3Kγ inhibitor 3-Methyladenine (3-MA) to TAMs. The system, PHNPs@DPA-S-S-BSA-MA@3-MA, effectively inhibited PI3Kγ expression, which in turn upregulated and activated the NF-κB p65 pathway. This molecular switch robustly polarized macrophages to a pro-inflammatory M1 phenotype, enhancing immunostimulatory genes while suppressing anti-inflammatory ones. In vivo, this TAM reprogramming remodeled the immunosuppressive tumor microenvironment by increasing infiltration of CD8^+^ and CD4^+^ T cells, B cells, and NK cells, while reducing Treg populations.

#### 4.1.2. CSF-1R Inhibitors

Colony-stimulating factor 1 receptor (CSF-1R) is a tyrosine kinase receptor expressed predominantly on macrophages and their progenitors, where it regulates survival, proliferation, differentiation, and recruitment [120]. In tumors, CSF-1/CSF-1R signaling is a central driver of the expansion, maintenance, and immunosuppressive programming of M2-like TAMs [121]. The Engagement of CSF-1R activates downstream pathways including JAK/STAT3, PI3K/AKT, ERK1/2, and NF-κB, collectively promoting transcriptional programs that enhance IL-10 production, suppress antigen presentation, elevate arginase-1 (Arg1), support angiogenesis, and inhibit effector T-cell responses [120,122,123]. This makes CSF-1R blockade one of the most validated and widely studied strategies for TAM depletion and repolarization [124,125]. Inhibition of CSF-1R can either deplete suppressive macrophages or repolarize them. However, while targeting the CSF-1 signaling pathway has the potential to improve glioblastoma therapy, systemic administration of CSF-1R inhibitors like BLZ945 faces significant challenges, including a narrow therapeutic window and off-target toxicities, which have limited their clinical success. To overcome this, a novel approach was developed using a hydroxyl dendrimer nanoparticle (D-BLZ) to deliver BLZ945 selectively to TAMs after systemic administration. This strategy localized the inhibition to the tumor site, where the dendrimer conjugate enabled sustained release of the inhibitor. The results demonstrated that a single dose of D-BLZ effectively repolarized TAMs away from their pro-tumor phenotype, increased cytotoxic T-cell infiltration, and ultimately prolonged survival in glioblastoma models, achieving superior efficacy compared to the free drug [126].

Building on the premise that TAM-targeted nanoparticles can safely reverse immunosuppression, the logical next step is to combine this strategy with agents that unleash the reactivated T-cells. In a subsequent study, Ramesh et al. tested this hypothesis by engineering a self-assembled lipid nanoparticle (LNP) system co-loaded with a CSF1R signaling axis and surface-functionalized with an anti-PD-L1 monoclonal antibody (mAb). This α-PDL1-CSF-LNP system serves a dual purpose: targeting PD-L1-expressing M2-like macrophages for drug delivery while simultaneously blocking the PD-1/PD-L1 checkpoint. The construct enhanced M2-to-M1 repolarization and the phagocytic index in vitro. In an aggressive melanoma mouse model, a suboptimal dose of α-PDL1-CSF-LNP achieved superior anti-tumor efficacy with minimal toxicity. Ex vivo analysis confirmed TAM repolarization and a marked increase in tumor-infiltrating CD8^+^ T cells, demonstrating a synergistic anti-tumor mechanism [127]. Rodriguez-Perdigon et al. developed ~400 nm polymersomes loaded with the selective CSF-1R inhibitor BLZ-945 to target TAMs in vitro. These CSF1Ri-loaded polymersomes are preferentially internalized by M2-like macrophages, enabling their repolarization toward a pro-inflammatory M1-like phenotype while minimizing cytotoxicity compared to free drug. In co-culture models of human monocyte-derived macrophages and MDA-MB-231 breast cancer cells, the nanoparticles effectively reduce immunosuppressive cytokines such as IL-10 and Arg1, enhance antigen presentation, and stimulate TNF-α and IL-12 production. This approach demonstrates the potential of nanocarrier-mediated CSF-1R inhibition for precise immunomodulation in tumor microenvironments [128]. Zhang et al. [129] reported a sequential tumor barrier–elimination strategy using an intratumoral ATP-responsive nanogel (B^BLZ-945^@PAC−PTX) to enhance chemoimmunotherapy. Upon reaching the tumor, the nanogel undergoes ATP-triggered collapse in perivascular regions, releasing BLZ-945-conjugated albumin to deplete TAMs. The shrunken PAC−PTX nanogel achieves deeper intratumoral penetration, inhibits CXCR4 signaling to reduce recruitment of immunosuppressive cells, and internalizes into tumor cells to induce cytotoxicity and T cell priming. This stepwise barrier elimination approach facilitates immune cell infiltration, potentiates antitumor immune responses, and represents a highly responsive strategy for combined chemoimmunotherapy.

#### 4.1.3. Toll-like Receptor (TLR) Agonists

Toll-like receptors (TLRs) are a family of pattern recognition receptors that detect pathogen-associated molecular patterns (PAMPs) and damage-associated molecular patterns (DAMPs), initiating innate immune responses [130]. Structurally, each TLR contains an extracellular ligand-recognition region, a single transmembrane segment, and an intracellular Toll/IL-1 receptor (TIR) domain that mediates downstream signaling. Cell-surface TLRs (TLR1, TLR2, TLR4, TLR5, TLR6, TLR10) primarily recognize microbial membrane components [131], whereas endosomal TLRs (TLR3, TLR7, TLR8, TLR9) detect nucleic acid ligands [132]. Upon ligand engagement, the TIR domain recruits adaptor proteins, including MyD88, TIRAP, TRAM, or TRIF, which assemble signaling complexes that activate NF-κB, IRF, and MAPK pathways, leading to the induction of pro-inflammatory cytokines or type I interferons [133,134]. Ultimately, enhancing antigen presentation, co-stimulatory molecule expression, and T-cell priming [135]. Endosomal TLR3 Agonists (e.g., poly I:C), TLR7/8 (e.g., imiquimod, resiquimod), and TLR9 (CpG-ODNs) can reprogram tumor-associated macrophages (TAMs) toward an M1-like phenotype. For instance, TLR3 stimulation with poly I:C promotes IFN-α/β–dependent upregulation of M1 markers (CD86, CD80, CD40) and pro-inflammatory cytokines, leading to tumor regression in vivo [136]. TLR7/8 agonists such as imiquimod and resiquimod drive NF-κB (and IRF)–mediated production of TNF-α, IL-12, and type I interferons [115]. Resiquimod (R848) has been shown to reprogram MDSCs/TAMs into tumoricidal macrophages in chemoresistant cancer models and to reduce tumor burden in murine lung cancer, while increasing M1 markers and systemic pro-inflammatory cytokines [137].

Rodell et al. [138] engineered a resiquimod derivative (R848-Ad) by conjugating an adamantane moiety, which enhances its affinity for cyclodextrin-based nanoparticles (CDNPs). This supramolecular strategy allowed efficient loading of R848-Ad, reduced systemic adverse effects, and maintained potent TAM-polarizing activity in a murine cancer model (MC38). Kim et al. [139] developed a lyophilizable nanoemulsion (NE) co-encapsulating the water-insoluble TLR7/8 agonists R837 and R848 to reprogram the tumor microenvironment and enhance cancer immunotherapy. Both agonists were dissolved in squalene with oleic acid and emulsified, producing NE(R837+R848) with a uniform size of approximately 147 nm. Local administration of NE(R837+R848) in murine melanoma and cervical cancer models promoted polarization of tumor-associated macrophages from an immunosuppressive M2-like phenotype toward a pro-inflammatory M1-like state, stimulated type I interferon production, inhibited tumor growth, and prolonged survival. Combination with immune checkpoint blockade (anti–PD-1/anti–PD-L1) further enhanced antitumor efficacy. Recently, an all-natural gelatin-based nanoemulsion was used to deliver the TLR7/8 agonist resiquimod (R848). Goswami et al. designed a cross-linked riboflavin-gelatin scaffold containing an oil core of eugenol that solubilized R848. These particles demonstrated excellent stability, low toxicity, and preferential uptake by M2-like macrophages, which they reprogrammed toward a pro-inflammatory M1-like phenotype [140]. More strikingly, a multi-functional Mn^2+^–CpG nanoadjuvant (MPN/CpG) was engineered by self-assembly of manganese ions, CpG (TLR9 agonists), and epigallocatechin gallate (EGCG). When delivered in vivo (B16 melanoma model), this formulation reprogrammed TAMs from an M2-like to M1-like state, reshaped the tumor microenvironment, and boosted infiltration of CD8^+^/CD4^+^ T cells and dendritic cells. These works illustrate that intelligently designed nanoparticle systems can both protect CpG, enhance its immune-stimulatory potency and selectively reprogram immunosuppressive macrophages in tumors [141].

#### 4.1.4. Cytokines Delivery

Cytokine-based immunotherapy has emerged as a powerful strategy for reshaping the tumor immune microenvironment, particularly through the reprogramming of TAMs. Key cytokines (example: IL-12, IL-15, and IFN-γ) can activate macrophages through canonical pathways, including JAK/STAT, NF-κB, and mTOR, driving enhanced antigen presentation, nitric oxide production, cytotoxic mediator release, and improved communication with NK cells and T lymphocytes [142,143,144,145,146]. However, systemic cytokine therapy faces limitations, including toxicity, short half-lives, and off-target effects [147,148]. Nanoparticles can overcome these challenges by delivering cytokines directly to the tumor site. Sun et al. [149] developed an acid-switchable Gal/IL-15@CaLN nanoparticle that co-delivers IL-15 and the TGF-β inhibitor galunisertib. These nanoparticles exhibit pH-triggered aggregation, enhancing tumor retention (>120 h) and enabling sustained cytokine and drug release in a CT26 colorectal tumor model. In vivo, Gal/IL-15@CaLN activated NK cells and CD8^+^ T cells while reprogramming TAMs toward an M1-like phenotype, increasing the CD80^+^/CD206^+^ ratio from 0.24 to 1.29. Mechanistically, IL-15 promoted NK cell activation via p-STAT5 and p-mTOR signaling, whereas galunisertib blocked TGF-β signaling, enhancing cytotoxic receptor expression and granzyme B/perforin secretion. By simultaneously boosting innate NK cell activity, adaptive T cell responses, and TAM repolarization, this platform exemplifies a synergistic and targeted strategy to potentiate antitumor immunity in cancer therapy. 

Shields et al. [150] developed “cellular backpacks,” a disk-shaped particulate system engineered to attach externally to macrophages without disrupting their migration or phagocytic behavior. The backpacks were fabricated using two layers of the biocompatible polymer poly(lactic-co-glycolic acid) (PLGA), sandwiching a polyvinyl alcohol (PVA) layer loaded with IFN-γ, enabling sustained and localized cytokine release directly on the macrophage surface. When applied to M2-skewed or TAMs, this configuration delivered prolonged pro-inflammatory cues that reprogrammed cells toward an M1 phenotype, markedly increased TNF-α and IL-12 secretion. In murine tumor models, macrophages equipped with these PLGA/PVA/IFN-γ backpacks exhibited improved intratumoral persistence and significantly reduced tumor growth, demonstrating a promising strategy for macrophage-based immunotherapy. 

**Figure 1:**
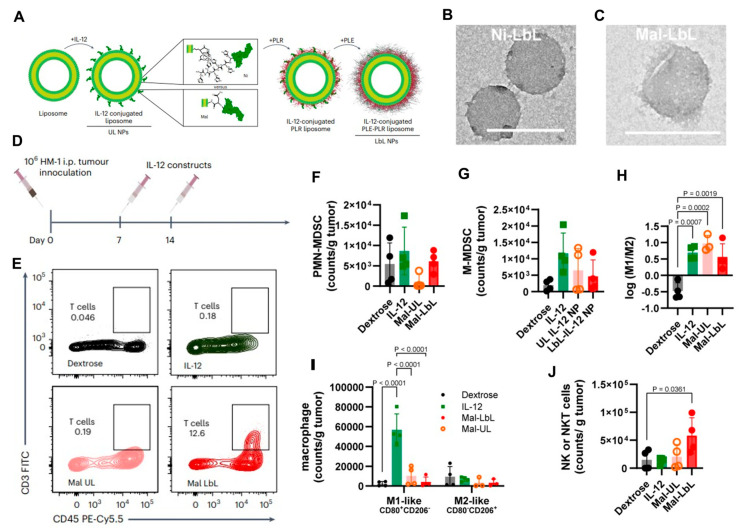
Layer-by-layer (LbL) nanoparticle–mediated cytokine delivery for immune modulation. (**A**) Schematic illustration of LbL nanoparticle (NP) construction, highlighting the use of maleimide (Mal) or nickel (Ni)-based linkers for surface conjugate IL-12 to the particle surface. Negatively stained Transmission Electron Microscopy (TEM) image of (**B**) Ni-LBL and (**C**) Mal-LBL nanoparticles; scale bars represent 200 nm. (**D**) Schematic representation of systemic cytokine delivery via the free IL-2 and IL-12 conjugated NP construct treatment on 0, 7, and 14 days at a dose of 20 μg in B6C3F1 mice (**E**–**J**) Flow analysis of tumor immune remodeling, including (**E**) T cell (**F**) Polymorphonuclear Myeloid-derived suppressor cell (PMN-MDSC) count (**G**) Monocytic myeloid-derived suppressor cell (M-MDSC) count (**H**) M/M2-like macrophage conversion ratio (**I**) M1- and M2-like macrophage total cell counts (**J**) NK cell count in tumor nodules. (Reprinted with permission from Ref. [152]. Copyright 2025, Springer Nature).

Cytokines such as IL-12 are potent drivers of macrophage activation (JAK/STAT pathway), boosting antigen presentation, cytotoxicity, and communication with adaptive immunity [151]. Pires et al. [152] developed polymer-coated nanoparticles for IL-12 delivery to address issues associated with free cytokine therapy, including rapid degradation and systemic toxicity. These nanoparticles were coated with a layer-by-layer (LbL) polymer system consisting of poly-L-arginine (PLR) and poly-L-glutamate (PLE) to enhance stability and bioavailability. In models of metastatic ovarian cancer, these nanoparticles provided sustained, localized IL-12 release within the tumor microenvironment, promoting T cell infiltration and activation, as well as enhancing the pro-inflammatory M1-like phenotype. Additionally, they reduced myeloid-derived suppressor cells, increased natural killer (NK) cell levels, and improved overall antitumor efficacy (Figure 1). Compared to free IL-12, the nanoparticle formulation significantly extended survival and widened the therapeutic window, emphasizing its potential as a safe and effective cytokine-based immunotherapy. Similarly, PLGA-DMMA-based pH-responsive nanoparticles carrying IL-12 were designed to remain stable at physiological pH but release IL-12 selectively within the mildly acidic tumor microenvironment. In a 3D macrophage-rich immuno-spheroid model, these nanoparticles successfully reprogrammed macrophages from an M2-like to an M1-like phenotype, as shown by increased CD86^+^ and decreased CD163^+^ expression. They also boosted interferon-gamma (IFN-γ) production while reducing the immunosuppressive cytokine IL-10 [153].

#### 4.1.5. RNA-Based Reprogramming of TAMs

**Figure 2:**
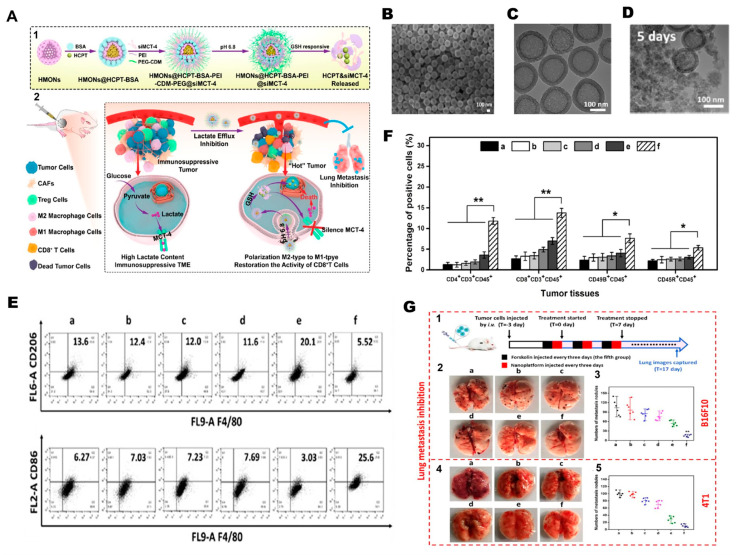
Cascaded-responsive nanoplatform for GSH-triggered drug release and immunogenic tumor reprogramming. (**A**) Schematic illustration showing the cascaded-responsive nanoplatform (HMONs@HCPT-BSA-PEI-CDM-PEG@siMCT-4) undergoing stimuli-triggered degradation to release HCPT and siMCT-4. HCPT induces tumor cell apoptosis and blocks lactate efflux, converting the immunosuppressive TME into a “hot” tumor. This strategy repolarizes TAMs from M2 to M1 and restores CD8^+^ T-cell activity, enhancing chemo-immunotherapy. (**B**) SEM image, (**C**) TEM images before, (**D**) TEM image after incubating the HMNOs for 5 days in a 10 mM GSH-containing buffer, demonstrating nanoparticle degradation. (**E**) Flow cytometry analysis showing Macrophage repolarization from M2-to-M1 conversion in 4T1 tumor tissues, (**F**) Flow cytometry analysis showing immune response (CD4^+^CD3^+^CD45^+^ helper T cells, CD8^+^CD3^+^CD45^+^ cytotoxic T cells, CD49B^+^CD45^+^NK cells, and CD45R^+^CD45^+^B cells) of 4T1 tumor tissues. (**G1**) Schematic illustration showing nanoparticle treatment and inhibition of lung metastasis nodules in Balb/c mice bearing the B16F10 tumor model and 4T1 tumor model. (**G2**) Lung metastatic nodules in B16F10 tumor-bearing mice. (**G3**) Average number and individual counts of lung metastatic nodules in BALB/c mice. (**G4**) Lung metastatic nodules in tumor-bearing mice. (**G5**) Average number and individual counts of lung metastatic nodules in BALB/c mice. Error bars represent mean ± SD for *n* = 6 (* *p* < 0.05, ** *p* < 0.01). (Reprinted with permission from Ref. [154]. Copyright 2020, American Chemical Society).

RNA therapeutics, including small interfering RNAs (siRNAs), microRNAs (miRNAs), and epigenetic modulators delivered as RNA or RNA-guided molecules, provide precise control over gene expression and intracellular signaling pathways that govern macrophage polarization. Li et al. [154] designed a cascade-responsive hollow mesoporous organosilica nanoplatform (HMONs) coated with a PEG–CDM shell to deliver siMCT-4, targeting monocarboxylate transporter-4, a key mediator of tumor lactate efflux. The system responds to intracellular GSH to trigger shell detachment, siRNA release, and efficient cytosolic delivery. Silencing MCT-4 reduced extracellular lactate accumulation, relieving metabolic immunosuppression within the tumor microenvironment and repolarizing TAMs from an M2 to a pro-inflammatory M1 phenotype. This metabolic rewiring enhanced antigen presentation and restored CD8^+^ T-cell cytotoxicity in vivo (Figure 2). In 4T1 and B16F10 models, the combined blockade of lactate efflux and chemotherapy markedly inhibited primary tumor growth, suppressed lung metastasis, and improved survival, demonstrating potent chemo-immunotherapeutic synergy via TAM metabolic modulation. Shobaki et al. [107] engineered a CL4H6-based lipid nanoparticle (LNP) platform incorporating a pH-responsive cationic lipid to deliver siRNAs to tumor-associated macrophages (TAMs) selectively. The LNPs encapsulated siSTAT3, a small interfering RNA targeting Signal Transducer and Activator of Transcription 3, a central driver of M2-like polarization and immunosuppressive cytokine signaling, together with siHIF-1α, which targets Hypoxia-Inducible Factor 1-alpha, a master regulator of hypoxia-driven angiogenesis. In a human tumor xenograft model, these CL4H6-LNPs were efficiently internalized by TAMs and achieved potent gene silencing. Dual STAT3/HIF-1α knockdown increased CD11b^+^ macrophage infiltration, promoted M1-like reprogramming, and reduced angiogenesis-associated gene expression. Collectively, this coordinated silencing strategy reversed TAM pro-tumoral functions and produced a measurable anti-tumor response. 

Liu et al. [155] developed reduction-responsive nanoparticles composed of poly(disulfide amide) (PDSA) and lipid-PEG for systemic co-delivery of Siglec-15 siRNA and IFN-γ. After intravenous administration, the nanoparticles efficiently accumulated in hepatocellular carcinoma (HCC) tumors and were readily internalized by tumor-associated macrophages (TAMs). Elevated intracellular glutathione levels triggered rapid nanostructure disassembly, enabling controlled release of IFN-γ to repolarize TAMs and enhance CXCL9-mediated T-cell infiltration, while siSiglec15 simultaneously silenced Siglec-15 to promote T-cell proliferation. In murine HCC models, this dual-cargo system markedly inhibited tumor growth and showed further therapeutic enhancement when combined with anti-PD-1 checkpoint blockade. Xia et al. [156] developed lipid nanoparticles (LNPs) to deliver Dicer-targeting siRNA specifically to tumor-associated macrophages (TAMs) in a mouse model of colorectal cancer liver metastasis. The LNPs protected siRNA in circulation and facilitated efficient TAM uptake and cytoplasmic release. Silencing Dicer disrupted miRNA biogenesis, downregulating immunosuppressive miRNAs such as miR-148a-3p and miR-1981-5p, and repolarizing TAMs toward a pro-inflammatory M1 phenotype. This enhanced antigen presentation, cytokine secretion, and cytotoxic T-cell recruitment. In vivo, treatment significantly reduced metastatic tumor burden and improved anti-tumor immunity. Taghavi-Farahabadi et al. [157] engineered M1 macrophage-derived exosomes (from LPS-activated RAW264.7 cells) to deliver siRNAs targeting SIRPα and STAT6 into M2-like TAMs. Electroporated exosomes repolarized M2 macrophages toward a pro-inflammatory M1 phenotype, enhancing phagocytosis and reducing migration, invasion, and proliferation of 4T1 breast cancer cells. When combined with anti-PD-L1 therapy, this approach further boosted anti-tumor macrophage functions, demonstrating a promising multi-dimensional strategy for reprogramming TAMs and improving immunotherapy efficacy.

MicroRNAs (miRNAs) are small non-coding RNAs that regulate gene expression post-transcriptionally and play critical roles in immune cell function, including macrophage polarization. Among them, microRNA-155 (miR-155) is a well-characterized pro-inflammatory miRNA in macrophages, upregulated in response to lipopolysaccharides or interferon-β and mediating inflammatory signaling via the NF-κB pathway [158,159]. Wu et al. [160] developed targeted lipid nanoparticles (LNPs) modified with an anti-SIRPα antibody to deliver miR-155 specifically into TAMs. The nanoparticles not only enabled efficient miRNA delivery but also blocked the SIRPα–CD47 “don’t-eat-me” signal, enhancing macrophage phagocytosis. In a B16F10 melanoma mouse model, miR-155 delivery repolarized TAMs toward a pro-inflammatory M1-like phenotype, increased TNF-α and IL-12 production, and promoted cytotoxic CD8^+^ T-cell infiltration, demonstrating a promising miRNA-nanoparticle approach for reprogramming TAMs and potentiating anti-tumor immunity. In addition to the studies discussed above, a range of comparable experimental findings is summarized in Table 1.

### 4.2. Material-Intrinsic Signaling

Nanomaterials frequently act as active biological signals, not just passive carriers. Their core composition, surface chemistry, size/shape, and charge can directly reprogram macrophage phenotype through several material-intrinsic mechanisms: (i) Fenton-type redox chemistry and metal ion release that elevates intracellular reactive oxygen species (ROS) and reactive nitrogen species (RNS; via iNOS/NO production) to promote pro-inflammatory (M1-like) transcriptional programs; (ii) autophagy modulation and lysosome-dependent signaling that changes cytokine secretion and antigen processing; and (iii) direct receptor and membrane interactions driven by surface ligands and charge that bias downstream signaling cascades (NF-κB, STATs). These intrinsic effects are highly dependent on particle chemistry and presentation. For example, iron-based particles commonly drive ROS-linked M1 responses [174], whereas some gold or peptide-coated gold nanoparticles skew macrophages toward tissue-repairing M2 phenotypes in a size-and ligand-dependent manner [175,176].

#### 4.2.1. Fenton-Type Redox Reactions

Iron oxide nanoparticles increase labile metal pools and catalyze ROS generation inside macrophages, activating MAPK/NF-κB pathways and upregulating TNF-α, IL-6, and iNOS, thereby favoring M1-like programs in many contexts. Polyaniline-coated iron oxide nanoparticles (Pani/γ-Fe_2_O_3_) have been shown to reprogram tumor-associated macrophages (TAMs) toward a pro-inflammatory, M1-like phenotype and to reshape the tumor microenvironment in a breast-cancer model. Treatment increased production of pro-inflammatory cytokines (e.g., IL-12p70, TNF-α, IL-6) and hydrogen peroxide, promoting tumor-cell apoptosis in vitro. In vivo, administration of these nanoparticles reduced 4T1 tumor weight and volume by roughly 50%, elevated CD86^+^ (M1) macrophages and natural killer (NK) cells, diminished neutrophil infiltration, and suppressed lung metastasis. These data highlight Pani/γ-Fe_2_O_3_ as an immunostimulatory nanomaterial capable of re-educating TAMs and inhibiting tumor progression without conventional drug loading [177]. In another study, Horvat et al. [178] showed that superparamagnetic iron oxide nanoparticles (SPION-CCPMs) can reprogram TAMs toward a pro-inflammatory, tumor-suppressive phenotype in a lung cancer model. Following phagocytosis, macrophages loaded with SPION-CCPMs secrete reactive nitrogen species (RNS) and inflammatory cytokines (e.g., TNF-α, IL-6, IL-1β), leading to oxidative stress and DNA damage in neighboring cancer cells, thereby reducing their proliferation and viability. In vivo, intratracheal instillation of SPION-CCPMs in tumor-bearing mice reshapes the immunosuppressive tumor microenvironment (TME), increases infiltration of cytotoxic CD8^+^ T cells, and delays tumor growth. Most notably, when administered after first-line tyrosine-kinase inhibitor therapy (e.g., Crizotinib), SPION-CCPM treatment significantly inhibits the regrowth of relapsing tumors. This establishes SPION-CCPMs as a promising iron-based nanomaterial capable of remodeling the TME and suppressing lung cancer relapse without additional chemotherapeutic or checkpoint-blockade drugs. Zanganeh et al. [179] demonstrated that Ferumoxytol, an FDA-approved ultrasmall superparamagnetic iron oxide nanoparticle (SPION), exerts a potent material-intrinsic antitumor effect through macrophage reprogramming and Fenton-driven oxidative stress. In co-cultures of MMTV-PyMT breast cancer cells and macrophages, ferumoxytol induced a pronounced 11-fold increase in hydrogen peroxide and a 16-fold increase in hydroxyl radical production, confirming the presence of active Fenton-type chemistry. This ROS burst significantly elevated caspase-3 activation in cancer cells, indicating enhanced macrophage-mediated cytotoxicity. Ferumoxytol-exposed macrophages also upregulated Th1-associated pro-inflammatory transcripts, reflecting a shift toward an M1-like phenotype. In vivo, ferumoxytol markedly inhibited the growth of subcutaneous adenocarcinomas and reduced lung cancer metastasis to the liver and lungs. Flow cytometry and histopathology confirmed increased infiltration of pro-inflammatory M1 macrophages in tumor tissues. Together, these findings establish ferumoxytol as a potent intrinsic immunomodulator capable of amplifying macrophage ROS production, promoting tumor cell apoptosis, and suppressing metastatic spread.

Ramkrishna Pal et al. [180] investigated the intrinsic properties of gold (AuNPs) and silver nanoparticles (AgNPs) and their antitumor potential using TAMs isolated from murine fibrosarcoma. Both AuNPs and AgNPs modulate ROS and RNS production, suppressing the antioxidant defenses of TAMs while maintaining ROS/RNS as second messengers to activate proinflammatory signaling cascades. This leads to downregulation of TNF-α and IL-10 and upregulation of IL-12. Consequently, TAMs are reprogrammed from an M2 (anti-inflammatory) to an M1 (pro-inflammatory) phenotype. The study highlights how noble metal nanoparticles can intrinsically modulate macrophages through oxidative stress mechanisms resembling Fenton reactions. Xu et al. [181] demonstrated that copper sulfide nanoparticles (CuS NPs) can reprogram tumor-associated macrophages (TAMs) to enhance antitumor activity in melanoma. The nanoparticles are internalized by macrophages and induce intracellular reactive oxygen species (ROS) production, partly via mitochondrial fission mediated by Dynamin-related protein 1 (Drp1). Elevated ROS activates proinflammatory signaling cascades, including the IKK-dependent NF-κB pathway, driving the polarization of TAMs from an M2-like, immunosuppressive state to an M1-like, pro-inflammatory phenotype. Reprogrammed macrophages exhibit enhanced phagocytosis and antitumor activity, and their adoptive transfer into melanoma-bearing mice results in tumor suppression and prolonged survival.

#### 4.2.2. Autophagy/Lysosome Signaling

Nanomaterials can also reprogram macrophages by altering autophagy and autophagic flux, thereby directly steering macrophage polarization. A recent study by Baimanov et al. [113] demonstrated that lanthanide-based metal oxide nanoparticles (LaNiO_3_, LNO), a perovskite-type metal oxide, possess intrinsic phosphatase-like activity that hydrolyzes intracellular ATP and dephosphorylates regulatory proteins, thereby inducing energetic stress in macrophages. The resulting ATP depletion activates AMP-activated protein kinase (AMPK), a central energy sensor, and triggers autophagy via the AMPK/mTOR pathway. LNO treatment increased LC3-II expression, and this accumulation was further enhanced in the presence of the autophagy-flux inhibitor bafilomycin A1, indicating elevated autophagosome synthesis independent of lysosomal degradation. In B16 tumor-bearing C57BL/6 mice, LNO-induced autophagy was associated with reduced M2-like macrophage populations and significant tumor suppression, highlighting autophagy modulation as a key material-intrinsic mechanism of macrophage reprogramming. Conversely, a study by Zhang et al. [182] showed that PEG-gold nanoparticles (PEG-AuNPs) inhibit autophagic flux in TAMs by inducing lysosomal alkalization and membrane permeabilization, impairing autophagosome–lysosome fusion and preventing autolysosome formation. This blockade of autophagy suppressed expression of M2-associated genes (e.g., Arg1, Mrc1/CD206, IL-10) while activating pro-inflammatory pathways, including NF-κB and MAPK signaling. PEG-AuNP–treated macrophages exhibited increased transcription of M1-related cytokines such as TNF-α, IL-12, IL-1β, and CXCL9/10, along with enhanced iNOS/NO production. In tumor-bearing mice, autophagy inhibition by PEG-AuNPs disrupted the immunosuppressive TME, enhanced infiltration of both CD3^+^CD4^+^ helper T cells and CD3^+^CD8^+^ cytotoxic T cells, and resulted in significant suppression of tumor growth. Together, these findings demonstrate that nanoparticle-mediated autophagy modulation, whether activation or inhibition, can be strategically leveraged to reprogram TAMs, where blocking autophagic flux shifts macrophages toward an M1-like, tumoricidal phenotype.

Similarly, semiconductor nanomaterials such as cadmium telluride quantum dots (CdTe-QDs) have been reported to modulate macrophage polarization via autophagy/lysosomal pathways. Wei et al. [183] demonstrated that low-dose CdTe-QDs induce M1 polarization in THP-1-derived macrophages, characterized by upregulated CD86, elevated NF-κB activity, and increased IL-1β secretion. Mechanistically, CdTe-QDs activate the mTOR–TFEB pathway, enhancing lysosomal signaling and autophagy, which drives the shift toward a pro-inflammatory, tumoricidal phenotype. Nevertheless, only a limited number of nanoparticle types have been studied in this context, underscoring the need for further investigation.

#### 4.2.3. Surface Chemistry and Receptor Engagement

Nanoparticle surface properties, including ligands, charge, hydrophobicity, and topography, strongly influence macrophage interactions and polarization by modulating both receptor engagement and protein-corona formation. For instance, a recent study using surface-functionalized mesoporous silica nanoparticles (MSNs) with different functional groups (neutral, negative, positive) demonstrated that surface chemistry significantly alters the composition of the protein corona and consequently changes how macrophages internalize the particles. Even MSNs with similar net surface charge exhibited distinct corona compositions and were internalized at different rates by macrophages [94]. Similarly, classical studies by Kurtz-Chalot et al. [184] have shown that surface functionalization of silica nanoparticles (e.g., amine or carboxyl groups, ±PEG) strongly affects uptake by macrophages, cytotoxicity, and secretion of pro-inflammatory mediators, with PEGylation reducing uptake and surface-functionalized (non-PEG) particles promoting uptake and inflammatory response. 

Beyond corona-mediated effects, specific surface ligands can directly engage macrophage receptors and bias polarization. In one example, Serrasqueiro et al. 2023 [185] reported that drug-free fucoidan/chitosan nanoparticles functionalized with mannose (or mannan) triggered activation of human THP-1-derived macrophages, leading to increased expression of the co-stimulatory marker CD86 and secretion of pro-inflammatory cytokines IL-1β, IL-6, and TNF-α, consistent with an M1-like phenotype. In another case, Hatami et al. [186] showed that mannose-decorated hybrid polymer/tannic-acid nanoparticles (MDNPs) exhibited mannose-dependent uptake by macrophages (U937 cells), indicating receptor-mediated internalization via the macrophage mannose receptor (CD206). These studies illustrate that ligand identity and density (e.g., mannose) on the nanoparticle surface can directly engage macrophage lectin receptors and modulate both uptake and downstream immune responses. Surface topography further influences macrophage activation. Wang et al. [187] showed that mannose-functionalized graphene oxide (MGO) utilizes CD206-mediated receptor targeting together with the intrinsically wrinkled, high-aspect-ratio surface morphology of graphene oxide to enhance membrane contact, promote receptor clustering, and facilitate selective uptake by M2-like TAMs and CD206-positive cancer stem cells. This synergy between ligand presentation and surface tomography contributes to M2-to-M1 repolarization, accompanied by increased TNF-α production and elevated ROS generation, which collectively remodel the immunosuppressive tumor microenvironment.

Functional Gadofullerene nanoparticles (GF-Ala) further exemplify carbon-based nanomaterial-mediated TAM modulation. The carbon cage and Ala functionalization polarize M2-like TAMs toward M1, upregulating TNF-α, CD86, and iNOS, downregulating IL-10, Arg-1, and CD206, and increasing CD8^+^ T-cell infiltration in tumors. These effects occur without additional chemotherapeutic or phototherapeutic agents, demonstrating that intrinsic material properties alone can remodel the tumor microenvironment and stimulate robust antitumor immunity in vivo [188]. In addition, Wu et al. [189] reported that multiwalled carbon nanotubes (MWCNTs) have been shown to prevent tumor metastasis by reprogramming M2-like TAMs toward a pro-inflammatory M1 phenotype. This polarization occurs through TLR4-mediated receptor activation, leading to enhanced production of pro-inflammatory cytokines and increased antitumor immune activity. A unique mechanism is observed with graphdiyne oxide (GDYO) nanosheets, which acquire an intracellular protein corona upon macrophage internalization, prominently featuring STAT3. Interactions between GDYO and STAT3, mediated by structural complementarity, hydrogen bonds, and salt bridges, drive the reprogramming of M2-like TAMs toward an M1 phenotype. This polarization enhances cytokine secretion, antigen presentation, and T-cell activation within the tumor microenvironment, all without additional drugs or external stimuli, underscoring intracellular corona formation as a novel material-intrinsic mechanism for immune modulation [190].

### 4.3. Combinatorial Multi-Pathway Platforms

**Figure 3:**
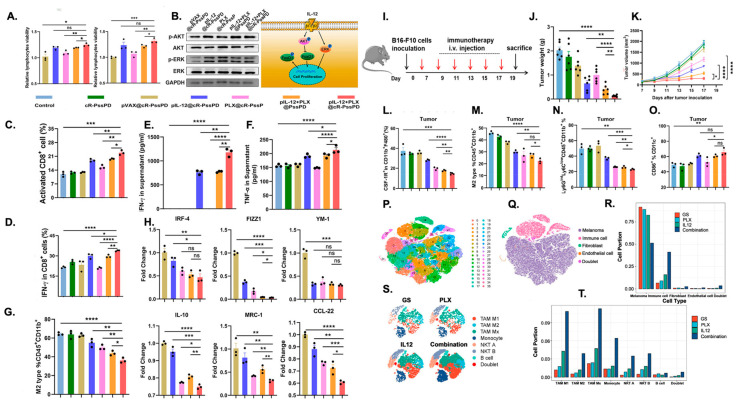
pIL-12+PLX@cR-PssPD–mediated lymphocyte activation and tumor immune reprogramming. pIL-12-driven immune activation via AKT/ERK signaling promotes antitumor immunity in vitro and in vivo. (**A**) Viability of lymphocytes after stimulation with tumor cell supernatant from different groups for 24 and 48 h. (**B**) Western blot showing the expression level of AKT/ERK pathway activation (p-AKT/AKT and p-ERK/ERK proteins) in lymphocytes after stimulation with tumor cell supernatant from different groups for 48 h. (**C**–**F**) Flow cytometry and cytokine analysis demonstrating CD8^+^ T-cell activation, including (**C**) Percentage of tumor-infiltrating CD8^+^CD69^+^ cell subset in the spleen and (**D**) CD8^+^IFN-γ^+^ lymphocytes’ subsets after 24 h of coculture with transfected tumor cells. (**E**) The secretion level of IFN-γ and (**F**) TNF-α from lymphocytes after coculturing with tumor cells for 48 h. (**G**) Flow cytometry analysis of CD45^+^CD11b^+^F480^+^CD206^high^ (M2 phenotype) cell subset. (**H**) Reverse transcription-polymerase chain reaction (RT-PCR) analysis of M2 phenotype-related genes. pIL-12+PLX@cR-PssPD restrained melanoma subcutaneous tumor growth in vivo. (**I**) Schematic illustration of the treatment regimen in a mouse model of B16-F10 subcutaneous tumor. (**J**) Tumor weights of all treatment groups (**K**) The curves of tumor volume changes of tumor-bearing mice. (**L**) The proportion of tumor-infiltrating CD11b^+^CSF-1R^+^ cell subset in the tumor. (**M**) The proportion of tumor-infiltrating CD45^+^CD11b^+^F480^+^CD206^+^ cell subset in the tumor. (**N**) The proportion of tumor-infiltrating CD11b^+^CD45^+^Ly6GintLy6Clow cell subset in the tumor. (**O**) The proportion of CD11c^+^CD86^+^ cell subset infiltration in the tumor. (**P**) T-distributed stochastic neighbor embedding (t-SNE) plots of distinct cell clusters color-coded by the annotated cell types in all samples (different colors indicated cells with different gene expression profiles detected by the Seurat package). (**Q**) The cells were divided into Melanoma, Immune cell, Fibroblast, Endothelial cell, and Doublet clusters. (**R**) Bar plot showing frequency of Melanoma, Immune cells, Fibroblast, Endothelial cell, and Doublets among different treatment groups. (**S**) Color-annotated major immune cell clusters (TAM M1, TAM M2, TAM Mx, Monocyte, NKT A, NKT B, B cell, and Doublet) distribution in individual treatment groups (The density of points represented the number of specific cell types). (**T**) Bar plot showing frequency of major immune cell types among different treatment groups. * *p* < 0.05; ** *p* < 0.01; *** *p* < 0.001; **** *p* < 0.0001; “ns”—no significant; (Reprinted with permission from Ref. [61]. Copyright 2024, American Chemical Society).

Combinatorial platforms intentionally pair two or more modalities, such as small molecules, nucleic acids, pattern-recognition agonists, physical stimuli, or material-intrinsic cues to engage complementary TAM biology simultaneously, such as inhibiting immunosuppressive signaling while activating innate danger pathways and modulating metabolism. These designs enhance the efficacy and durability of TAM reprogramming by producing synergistic intracellular signaling, concentrating complementary payloads within TAMs to minimize systemic toxicity, and leveraging material properties (e.g., redox activity, photothermal/sonodynamic capacity, membrane mimicry) to amplify immune stimulation independent of cargo. For example, Hu et al. [61] successfully remodeled the tumor microenvironment and enhanced antitumor immunity by codelivering an IL-12–expressing plasmid and the CSF-1R inhibitor PLX3397 using a GSH-responsive, cRGD-targeted nanocarrier containing disulfide bonds for reduction sensitivity. Encapsulation of pIL-12 promoted lymphocyte proliferation and activation, reflected by increased CD4^+^/CD8^+^ T-cell activation, elevated phosphorylated AKT and ERK signaling, and higher IFN-γ and TNF-α secretion. These immune shifts reduced CD45^+^CD11b^+^F4/80^+^CD206^high^ M2 macrophages and drove M1 repolarization. In a melanoma model, pIL-12+PLX@cR-PssPD significantly decreased tumor volume, reduced MDSCs, and increased CD11c^+^CD86^+^ dendritic cells (Figure 3). The combination broadly reshaped innate and adaptive immunity, boosting T-cell activity, NK cytotoxicity, and antitumor cytokine production, leading to significantly delayed tumor growth and metastasis, improved survival, and durable antitumor immunity in treated mice. In addition to purely immune-targeted approaches, combinatorial platforms have integrated conventional therapies such as radiotherapy with immune modulators to enhance TAM reprogramming. Ni et al. [191] developed an Hf-MOF nanoplatform codelivering a TLR-7 agonist (IMD) and an anti-CD47 antibody (αCD47). The Hf-MOF sensitizes tumors to radiotherapy, inducing immunogenic cell death, while IMD and αCD47 promote M2-to-M1 TAM polarization, dendritic cell maturation, and T-cell activation. This combination amplified antitumor immunity and produced durable tumor regression in vivo.

Photothermal therapy (PTT) induces localized hyperthermia that disrupts cell membranes, denatures proteins, and damages DNA, leading to apoptosis and the release of tumor antigens. PTT also triggers immunogenic cell death (ICD), enhancing DAMP release and promoting systemic antitumor immunity. Recent work by Deng et al. [192] demonstrated this synergy using a FeS_2_@COF nanocarrier, which integrates pyrite (FeS_2_) nanoparticles within a covalent organic framework (COF) to enable multimodal therapy. The FeS_2_ core provides intrinsic chemodynamic activity, while the COF enhances stability and photothermal conversion. Under NIR irradiation, the platform produces coordinated photothermal, chemodynamic, and thermodynamic tumor destruction, generating ROS and localized heat. This combined oxidative and thermal stress remodels the tumor immune microenvironment by reprogramming M2 tumor-associated macrophages toward a pro-inflammatory M1 phenotype, thereby promoting an antitumor immunity. Overall, the system achieves potent, immune-supported tumor suppression in vivo. Similarly, mitochondrial-targeted melanin@mSiO_2_ yolk-shell nanostructures have been developed for NIR-II-driven photo-thermal-dynamic/immunotherapy, achieving efficient M2-to-M1 TAM repolarization and enhanced antitumor immunity. Zhang et al. [193] also reported natural melanin nanoparticles (AIPH-MS-CTPP) that mediate TAM reprogramming through thermal and dynamic stimuli, highlighting the potential of melanin-based nanoplatforms to synergize material-intrinsic properties with phototherapy-induced immune modulation. Xiaoqing Ren et al. [194] developed magnetic nanoclusters (MNCs) composed of superparamagnetic iron oxide nanoparticles assembled into clusters, with a biocompatible coating that ensures stability and tumor accumulation. These MNCs serve a dual function: their iron-based core provides intrinsic redox activity that can modulate macrophage signaling, while the clustered structure enables efficient photothermal conversion under NIR irradiation. In vitro and in vivo, MNCs reprogram M2 tumor-associated macrophages toward M1, enhance cytokine secretion, and promote T-cell activation. When combined with PTT, the platform induces apoptosis, immunogenic cell death, and suppresses tumor growth and metastasis. Similarly, Zhao et al. [195] reported an iron-based MOF loaded with Capmatinib (Fe-MOF/Cap) that, combined with sublethal radiofrequency ablation (iRFA), repolarizes M2 TAMs, downregulates PD-L1, enhances CD8^+^ T-cell infiltration, and inhibits HCC lung metastasis via the c-MET/STAT3/VEGF pathway. Building on the ICD paradigm, Zhang et al. [196] designed ROS-responsive human serum albumin (HSA)-based PEG/IL12-IA nanoparticles incorporating indocyanine green (ICG, arginine, and IL-12. Under laser irradiation, ICG-generated ROS induce ICD in tumor cells while simultaneously triggering nanoparticle dissociation and controlled IL-12 release. Phototherapy-induced ICD drives dendritic cell maturation and strong T-cell priming, whereas ROS-responsive IL-12 release reprograms immunosuppressive M2 TAMs toward a pro-inflammatory M1 phenotype. In parallel, arginine metabolism enhances nitric oxide production, further relieving immunosuppression. These coordinated innate and adaptive immune responses significantly inhibited tumor growth, prolonged survival, and delayed metastasis in 4T1-bearing mice.

Graphene oxide (GO)-mediated PTT provides another example of physical-stimulus-driven TAM modulation. Carbon-based nanomaterials, as representative nonmetallic nanomaterials, are capable of inducing inflammatory responses, which can contribute to M2-to-M1 TAM repolarization and enhance the efficacy of photothermal cancer therapy. Deng et al. [197] demonstrated that polyethylene glycol-modified GO (GO-PEG) under near-infrared irradiation not only ablates tumor cells via photothermal heating but also reprograms M2-like TAMs toward a pro-inflammatory M1 phenotype. Conditioned medium from these re-polarized macrophages reduced tumor cell migration and invasion in vitro, and in vivo GO-PEG PTT significantly inhibited tumor growth, highlighting the contribution of TAM repolarization to therapeutic efficacy.

**Figure 4:**
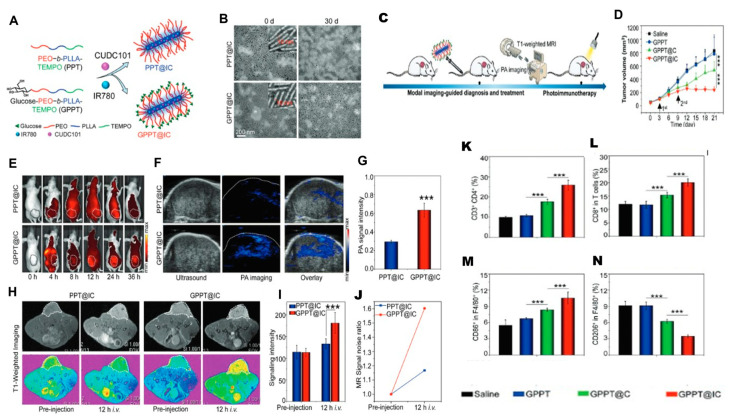
Glucose-based radical copolymer for MRI-guided photoimmunotherapy, combining imaging-guided phototherapy with immune activation to remodel the tumor microenvironment and enhance anticancer efficacy. (**A**) Schematic overview of the fabrication process of the copolymer micelles co-loading IR780 and CUDC101. (**B**) Representative TEM micrographs and DLS size distributions of PPT@IC and GPPT@IC at initial preparation and after 30 days of storage, demonstrating their morphological stability over time. In vivo evaluation: Biodistribution, tumor targeting, and therapeutic activity of glucose-modified GPPT@IC micelles were assessed in murine tumor models. (**C**) Timeline of the imaging-guided photoimmunotherapy protocol using GPPT@IC. (**D**) Tumor-volume progression in mice treated with saline, blank GPPT polymers, GPPT@C, or dual-loaded GPPT@IC, showing the superior antitumor efficacy of GPPT@IC. (**E**) Whole-body fluorescence imaging of Hep1-6 tumor–bearing nude mice at serial time points following intravenous administration of PPT@IC or GPPT@IC, highlighting enhanced tumor localization with glucose modification. (**F**,**G**) Photoacoustic images of tumors 12 h after injection of PPT@IC or GPPT@IC, further confirming improved tumor accumulation of GPPT@IC. (**H**) T1-weighted MRI used to track in vivo targeting and retention of PPT@IC and GPPT@IC micelles. (**I**) Quantitative T1-MRI signal intensity within the tumor before injection and 12 h post-injection. (**J**) Tumor-to-normal tissue MRI signal-to-noise ratio, illustrating significantly higher contrast in the GPPT@IC group (*** *p* < 0.001). (**K**–**N**) Flow-cytometric analysis of immune populations in the spleen of Hep1-6 tumor–bearing C57BL/6 mice following the respective treatments, including CD4^+^ T cells (**K**), CD8^+^ T cells (**L**), M1-like macrophages (**M**), and M2-like macrophages (**N**), demonstrating systemic immune activation elicited by GPPT@IC-mediated photoimmunotherapy. (Reprinted with permission from Ref. [199]. Copyright 2021, John Wiley and Sons).

Photodynamic therapy (PDT) offers precise tumor eradication through photosensitizers and light-triggered generation of reactive oxygen species (ROS), which oxidize cellular proteins and lipids [198]. Beyond direct cytotoxicity, PDT stimulates immunogenic cell death (ICD), triggers acute inflammation, releases tumor-specific antigens, and activates dendritic cells, collectively priming tumor-specific T cells. Notably, PDT-induced p53 activation has been shown to contribute to the repolarization of M2-like TAMs toward a pro-inflammatory M1 phenotype. Expanding combinatorial PDT strategies, Cheng et al. [199] developed a glucose-terminated radical copolymer (glucose-PEO-b-PLLA-TEMPO) nanoplatform co-loaded with a photosensitizer (IR780) and a CD47 inhibitor (CUDC101), enabling MRI-guided tumor accumulation (Figure 4). Upon light-triggered PDT, IR780 generates ROS while CUDC101 blocks CD47, synergistically promoting M2-to-M1 TAM repolarization, enhancing dendritic cell maturation and T-cell activation, and converting immunosuppressive “cold” tumors into immunoresponsive “hot” tumors. 

Sonodynamic therapy (SDT) is an emerging modality that employs acoustic sensitizers and focused ultrasound to generate ROS, inducing direct tumor cell death and cavitation-mediated membrane damage [200]. Recent advances have demonstrated the potential of combinatorial SDT platforms to simultaneously remodel the tumor microenvironment and reprogram TAMs. For example, Wang et al. [201] reported macrophage membrane-camouflaged SIM@TR-NPs, coated with engineered macrophage membranes overexpressing Siglec-10 and loaded with a sonosensitizer and immune adjuvant. These nanoparticles block “don’t eat me” signals, repolarize TAMs toward the pro-inflammatory M1 phenotype, and, upon ultrasound irradiation, trigger immunogenic cell death, enhancing antitumor immunity in ovarian cancer models. Similarly, Haiqin Liao et al. [202] reported a biomimetic nanoplatform (IR780/MnO_2_@PLGA@cell-membrane-PEP20) combining SDT, ferroptosis induction, and CD47 blockade. The MnO_2_ core depletes glutathione, triggering lipid peroxidation and ferroptosis, while IR780 generates ROS under ultrasound to amplify oxidative stress. The CD47-inhibitory peptide enhances macrophage phagocytosis, repolarizes M2 TAMs to M1, induces immunogenic cell death, and stimulates dendritic cell and cytotoxic T-cell activation. In a complementary strategy, Ming Li et al. [203] developed a folic-acid–modified, ultrasound-responsive nanocarrier (FA-PFNB-SIRPα siRNA) co-delivering SIRPα-targeting siRNA and Fe_3_O_4_ nanoparticles to TAMs in NSCLC. The interaction between SIRPα on macrophages and CD47 on cancer cells activates the “eat-me-not” signaling pathway, inhibiting phagocytosis. Under ultrasound, FA-PFNB-SIRPα siRNA suppressed SIRPα expression, promoted TAM phagocytosis, shifted M2-like macrophages toward a pro-inflammatory M1 phenotype, enhanced T-cell infiltration and activation, and significantly inhibited tumor progression. These multi-pathway strategies synergistically enhance antitumor efficacy, improve systemic safety, and convert immune “cold” tumors into “hot” tumors, highlighting SDT-based combinatorial platforms as a promising avenue for durable TAM-targeted immunotherapy. To integrate the diverse nanoplatform designs and immunomodulatory mechanisms discussed in Section 4 and Section 5, representative systems targeting tumor-associated macrophages (TAMs) and/or dendritic cells, along with their immune activation outcomes, are compared and summarized in Table 2.

## 5. Nanomaterials for Innate Immunity Activation and Vaccination

Nanomaterials have become indispensable tools for cancer immunotherapy due to their ability to engage and modulate both the innate and adaptive immune systems with precision. Dendritic cells (DCs), as the most potent antigen-presenting cells (APCs), represent a central target for nanoparticle-based vaccine strategies because they orchestrate T-cell priming and shape the quality of anti-tumor immunity. Table 3 summarizes ligand-decorated nanoparticles targeting TAMs, DCs, and other immune cells, while Table 4 highlights nanomaterials co-targeting TAMs and DCs for synergistic cancer immunotherapy. Nanoparticles can encapsulate, protect, and deliver diverse biomolecular cargoes. Nanoparticles can be engineered to mimic the size, charge, and multivalent signaling properties of pathogens, enabling them to naturally engage innate immune pathways. Moreover, their ability to protect fragile cargos such as RNA [207,208,209,210,211,212,213], peptides [214], plasmids [215], or adjuvants from enzymatic degradation allows for enhanced bioavailability and controlled release. This enables researchers to coordinate antigen delivery with the activation signals required to initiate potent anti-tumor immunity, enhancing antigen uptake, processing, and cross-presentation. These platforms allow antigens to be shielded from premature degradation and trafficked through the endosomal–lysosomal pathway, where cross-presentation machinery is activated. Additionally, nanoparticles can be conjugated with adjuvants such as TLR agonists, STING agonists, or inflammasome activators that enhance DC maturation and promote pro-inflammatory responses [205,216,217,218,219,220,221].

A major advantage of nanomaterials lies in their ability to modulate both arms of the immune system simultaneously. Alongside targeting DCs, nanoparticles have been leveraged to influence macrophage behavior within the tumor microenvironment. Macrophages are critical innate immune cells responsible for phagocytosis, antigen processing, and initiating inflammatory responses. Several nanoparticle strategies have been developed to target Macrophages. These include delivering immunostimulants or immune checkpoint inhibitors (ICIs) to reverse their suppressive phenotype [222,223], packaging cancer cell-derived debris to promote antigen availability, and engineering macrophage- or tumor-mimicking nanoparticles [224,225,226,227,228,229,230] that redirect innate signaling pathways. By engineering hybrid nanoparticle systems to co-deliver antigens and immune-stimulatory cues, researchers are able to mimic the fundamental steps of natural immune activation and generate robust, sustained cytotoxic T-cell responses [231,232,233,234]. Nanomaterials can simultaneously enhance DC-mediated priming and reverse TAM-driven immunosuppression, ultimately promoting a coordinated and sustained anti-tumor immune response.

### 5.1. DC Activation and Vaccination

Dendritic cells (DCs) act as master regulators of adaptive immunity by integrating signals from the innate immune system and presenting processed antigens to T cells. Because DCs determine the strength, phenotype, and durability of T-cell responses, they are ideal targets for nanoparticle-mediated vaccination strategies. However, tumor-driven immunosuppression severely limits DC activation, antigen processing, and migration to lymphoid organs. Nanomaterials offer a solution by delivering tumor antigens and immunostimulatory molecules in a controlled and synergistic manner, thereby enhancing DC maturation and promoting efficient cross-presentation. Nanoparticle-based cancer vaccination strategies have therefore evolved to not only deliver tumor antigens, but also to modify the local immune landscape in ways that maximize DC functionality. These platforms enable the deliberate coordination of innate activation and adaptive priming improving antigen presentation, elevating costimulatory signaling, and ultimately enhancing cytotoxic T-cell infiltration into tumors. Together, these innovations represent a significant advancement in the design of cancer vaccines capable of overcoming traditional barriers such as poor antigen stability, weak DC stimulation, and tumor-driven immunosuppression.

#### 5.1.1. Nanoparticle as a Precision Antigen and Adjuvant Co-Delivery Systems

A defining advantage of nanomaterials in cancer vaccination is their ability to co-deliver antigens with immunostimulatory adjuvants. Precise co-delivery ensures that DCs receive both the antigenic signal and the maturation cues necessary to activate cytotoxic T lymphocytes (CTLs). Effective activation of antitumor immunity requires the coordinated integration of multiple immunological signals, each of which governs a distinct step in dendritic cell (DC) maturation and T-cell priming. Dendritic cells rely on external stimuli to transition from an immature sentinel state to fully activated antigen-presenting cells capable of initiating adaptive immunity.

This maturation process is triggered when DCs recognize external signals including External pathogen and danger signals (pathogen-associated molecular patterns (PAMPs) or damage-associated molecular patterns (DAMPs)), Inflammatory and environmental signals (inflammatory signaling pathways and inflammasome cascades), T cell-derived signals (major histocompatibility complex (MHC) class I and II molecules, costimulatory ligands (CD80, CD86, CD40), and chemokine receptors) driving the upregulation of key surface molecules required for T-cell priming, mediating migration to draining lymph nodes and initiating robust, durable T-cell immunity. Importantly, these same activation cues can be precisely engineered into nanomaterial platforms. By incorporating these signals into nanoparticles, researchers can trigger controlled DC maturation, promote efficient antigen processing, and enhance DC–T cell communication. This strategic integration allows nanoparticles not only to deliver antigens but also to supply the full spectrum of activation signals necessary for robust immune priming and durable antitumor responses.

**Figure 5:**
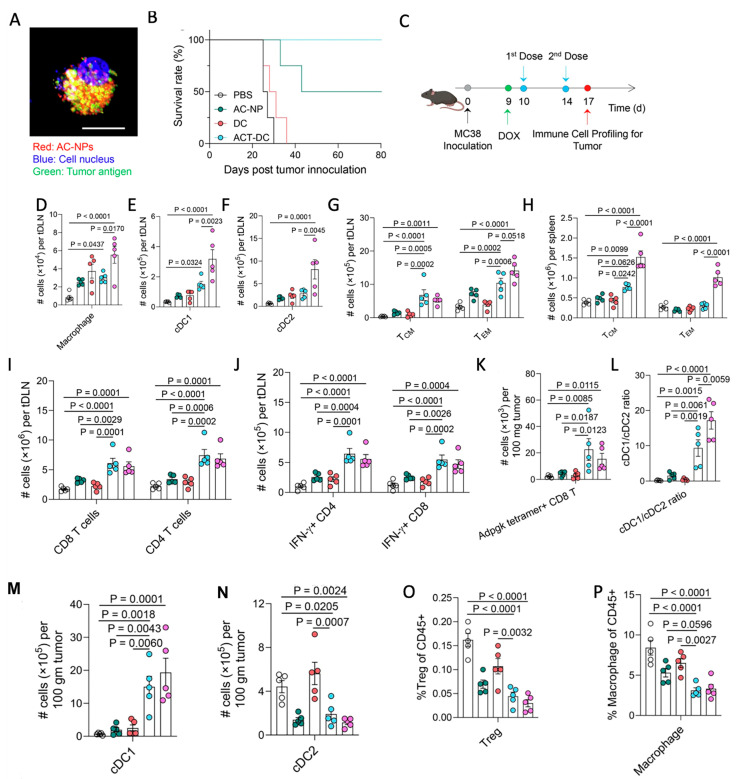
AC-NP–mediated tumor antigen delivery and cDC1-driven T-cell priming enhance antitumor immunity. (**A**) CLSM image showing AC-NP-assisted tumor antigen delivery into cDC1s after 4 h incubation. Scale bar: 10 μm. Representative image from three independent experiments. C-NPs efficiently activated cDC1s. (**B**) Survival curve of mice treated with ACT-DC or control formulations. (**C**) Schedule of the study to profile immune cells in the tumor. (**D**–**H**) Number of different immune cells in the tDLN of mice treated with ACT-DC or control therapies, including macrophages (**D**), cDC1 (**E**), cDC2 (**F**), Number of memory CD8 T cells in the tDLN (**G**) and spleen (**H**) of mice receiving the ACT-DC or control therapies. (**I**–**P**) Number of different immune cells in the tumor of mice treated with ACT-DC or control therapies, including the number of CD8/CD4 T cells (**I**) and IFN-γ expressing CD8/CD4 T cells (**J**), and the number of Adpgk tetramer-positive CD8 T cells (**K**) in the tumor. O-Q Profiles of cDC1s and cDC2s in the tumor. The cDC1/cDC2 ratio in the tumor (**L**), number of cDC1s (**M**), and cDC2s (**N**) in the tumor. (**O**,**P**) Profiles of Tregs and macrophages in the tumor. Percentage of Tregs (R) and macrophages (S) in the tumor following different treatments. (Reprinted with permission from Ref. [204]. Copyright 2025, Springer Nature).

Nanoparticles have been designed to sustain T cell activation by enabling continuous antigen stimulation through artificial APC-mimetic nanoparticles. For example, Chao et al. [204] designed polymer-based composite antigen-capturing nanoparticles (AC-NPs) using acid-ended poly(lactic-co-glycolic) acid (PLGA) and polyethylenimine (PEI) to allow for successful in situ immunization (Figure 5). Using these materials allowed for proper functioning to capture and deliver antigens; PLGA not only induced hydrophobicity to the NP, allowing for encapsulation of polyinosinic–polycytidylic acid (PIC), a toll-like receptor 3 (TLR3) agonist, but the PEI contributed positive surface charge, allowing for capture of tumor antigens through both electrostatic and hydrophobic interactions. Using CD103^+^ cDC1s from bone marrow as the model, they showed that AC-NP enhances the delivery of tumor antigens, shown through increased uptake of tumor proteins by cDC1s and substantial co-localization of tumor lysate proteins with AC-NPs inside cDC1s. This ability to efficiently capture tumor proteins and DAMPs, while enhancing tumor antigen delivery to and presentation on cDC1s, promoted CD80^+^ CD86^+^ double-positive activated cDC1s.

Transferring the AC-NP with or without adoptive transferred DCs showed enhanced trafficking to tDLNs, resulting in the presence of activated, antigen-carrying cDC1s in the tDLNs. This led to superior migratory capacity of CD103^+^ cDC1s toward tDLNs and their stronger antigen-presenting capabilities, in situ to tDLNs, and, in turn, initiate a cascading systemic antitumor immune response. Combining AC-NP with the adoptively transferred cDC1s significantly increased the number of innate immune cells in tDLNs, including macrophages, cDC1s, and cDC2s while also elevating the number of both central memory and effector memory CD8^+^ T cells in the tDLNs. By reshaping the initiation of the immune response, this was able to turn an immunologically “cold” tumor into a “hot” tumor represented by significantly increased the infiltration of CD4 and CD8 T cells, including Th1 cells, effector CD8 T cells (IFN-γ + CD8 T cells, Granzyme B+ CD8 T cells, and perforin+ CD8 T cells), while also significantly changing the frequency of immunosuppressive cells, including Tregs and macrophages, within the tumor leading to a robust tumor regression alone and in combination with the adoptively transferred DCs.

Beyond improving antigen uptake, next-generation nanoparticle strategies focus on directly enhancing dendritic cell (DC) activation to drive stronger immune responses. These platforms target multiple mechanisms of DC function, including increasing antigen delivery for more efficient processing, promoting DC maturation, and enriching lysosomal pathway-associated genes that support MHC II presentation. They also boost cross-presentation machinery for optimal CD8^+^ T-cell priming and counteract immunosuppressive signals within the tumor microenvironment [235]. Together, these approaches amplify DC activation and significantly strengthen downstream adaptive immunity. Nanomaterial-based delivery systems are uniquely positioned to orchestrate these signals with high precision. By supplying these essential cues in a controlled, spatiotemporally aligned manner, precision nanocarriers dramatically amplify immune activation and help overcome the intrinsic suppressive barriers of the tumor microenvironment.

#### 5.1.2. Reprogramming Tumor-Associated Macrophages to Support DC Function

Although DCs play a central role in adaptive activation, macrophages are the primary innate cells responsible for antigen uptake in solid tumors. Macrophages rapidly sense and respond to infection, injury, and tissue stress. Through phagocytosis, degradation of pathogens and dying cells, secretion of inflammatory mediators, and coordination of tissue repair, macrophages bridge innate and adaptive immunity. Their activation state is shaped by a complex network of environmental cues, including cytokines, chemokines, growth factors, metabolic signals, stromal interactions, and transcriptional regulators that collectively guide their functional phenotype. Classically activated M1 macrophages produce pro-inflammatory cytokines, generate reactive oxygen and nitrogen species, and promote antigen availability, whereas alternatively activated M2 macrophages support tissue repair but often contribute to tumor progression by suppressing immune activation. In solid tumors, macrophages are frequently skewed toward this M2-like tumor-associated macrophage (TAM) phenotype, limiting antigen presentation, dampening dendritic cell (DC) maturation, and impairing effective T-cell priming. This has inspired growing interest in using nanomaterials to reprogram TAMs toward an M1-like, inflammatory state that supports antigen release, cross-priming, and enhanced communication with DCs.

By understanding the molecular factors that drive macrophages toward either inflammatory or suppressive states, researchers can design nanomaterial-based strategies to therapeutically reprogram TAMs. Nanoparticles can be engineered to deliver inhibitors of immunosuppressive signaling pathways, block “don’t-eat-me” signals, or encapsulate immunostimulatory agents that promote M1-like polarization. Redirecting macrophages toward a pro-inflammatory, anti-tumor phenotype increases phagocytic clearance of tumor cells, enhances antigen release, and strengthens macrophage–DC communication. This macrophage reprogramming not only improves local innate immunity but also establishes the conditions necessary for effective DC maturation and subsequent activation of cytotoxic T-cell responses.

**Figure 6:**
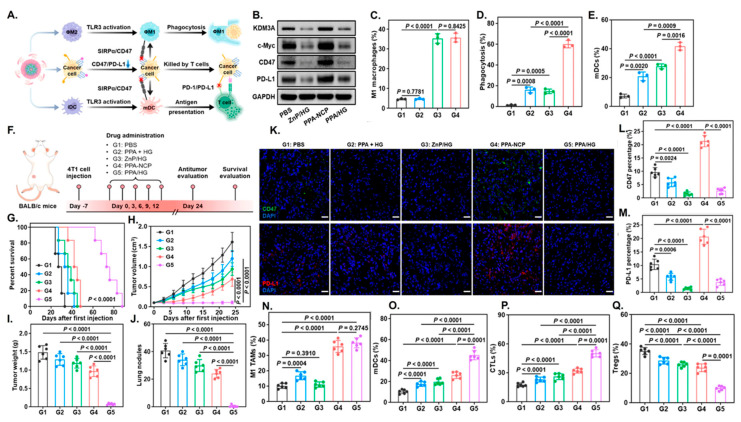
PPA/HG elicits innate and adaptive antitumor immunity. (**A**) Schematic illustration of PPA/HG-mediated antitumor immunity: PPA/HG reduces CD47 and PD-L1 expression in cancer cells and activates the TLR3 signaling pathway in macrophages and DCs, thereby enhancing macrophage phagocytosis, DC maturation, T cell proliferation, and activity. (**B**) Western blot analysis of KDM3A, c-Myc, CD47, and PD-L1 protein levels of 4T1 cells after indicated treatments. (**C**) Quantification of M1-like macrophages after indicated treatments. Flow cytometric quantification (**D**) of macrophage phagocytosis of 4T1 cells after indicated treatments and mature DCs (mDCs) after indicated treatments (**E**) PPA/HG treatment of orthotopic 4T1 tumors. (**F**) Schematic illustration of the experimental schedule. (**G**) Survival curves of the treated mice (**H**) Tumor growth curves of the treated mice, average weights (**I**) and the average numbers of lung nodules in the dissected 4T1 tumors treated mice (**J**). (**K**–**M**) CD47 (green) and PD-L1 (red) immunofluorescence staining of the 4T1 tumors (scale bars: 50 μm) (**K**) along with the quantitative analysis of CD47 (**L**) and PD-L1 (**M**) fluorescence signal quantified by ImageJ. (**N**–**Q**) Flow cytometry results of M1-like macrophage (F4/80^+^CD86^+^) in 4T1 tumors (**N**), mDCs (CD11c^+^CD80^+^CD86^+^) in TDLNs (**O**), CTLs (CD3^+^CD8^+^) in tumors (**P**), and Tregs (CD4^+^FOXP3^+^) in tumors (**Q**). (Reprinted with permission from Ref. [205]. Copyright 2025, American Chemical Society).

To further expand upon this activity, researchers have developed nanoparticles that can further modulate the immune compartment by targeting the activation and costimulatory signals required for a successful innate immune response initiation. Liu et al. [205] generated a PPA/HG, comprising polyinosinic: polycytidylic acid (PPA) in the core and a cholesterol-conjugated prodrug of 3-(hydroxyolinoyl) glycine (HG) on the shell, a combination of a TLR3 agonist and a KDM3A inhibitor for potent cancer immunotherapy. By encapsulating PPA/HG, they were not only able to increase the half-life of this drug, but they were also able to increase the accumulation in the tumors. By efficiently delivering this combined PPA/HG to the tumor, they were able to effectively downregulate CD47 and PD-L1 in tumors while activating toll-like receptor 3 in dendritic cells and tumor-associated macrophages to promote cell maturation and macrophage repolarization. In vivo, delivery of the PPA/HG markedly suppressed tumor growth of an orthotopic 4T1 tumor model, leading to significantly prolonged median survival. This therapeutic efficacy was attributed to the immunological effects of the PPA/HG acting on both the cancer cell and immune cell compartments in the in vivo system (Figure 6). The tumors were shown to effectively downregulate CD47 and PD-L1 expressions in 4T1 tumors while promoting infiltration and activation of effector T lymphocytes (CD45^+^ lymphocytes), Tumor-infiltrating macrophages (F4/80^+^CD86^+^CD206^−^ M1), DC maturation in TDLN (CD11c^+^CD80^+^CD86^+^), CTLs (CD3^+^CD8^+^), meanwhile decreasing the population of immunosuppressive regulatory T cells Tregs (CD4^+^FOXP3^+^). These results demonstrate that PPA/HG greatly enhances the infiltration and activation of CTLs in 4T1 tumors while efficiently relieving the immunosuppressive tumor microenvironment. Collectively, these findings highlight that the rational design of nanoparticles capable of modulating the development and phenotype of the tumor microenvironment (TME) can overcome immunosuppressive barriers by effectively activating both innate and adaptive immune responses. This synergistic immunomodulation ultimately promotes a robust systemic adaptive immunity elicited by the nanoparticle-based therapy. Nanoparticles are being generated to guide innate cells toward anti-tumor activity by restoring macrophage phagocytic capacity, thereby increasing antigen release and indirectly boosting DC activity. By reshaping macrophage behavior within the tumor microenvironment (TME), these approaches amplify the availability of tumor antigens and facilitate interactions with DCs, ultimately improving downstream T-cell priming.

#### 5.1.3. Coordinating Innate and Adaptive Immunity Through Nanomaterial Design

A major strength of nanomaterials is their ability to orchestrate multiple immune processes simultaneously [236,237,238,239,240,241,242]. By combining antigen delivery, macrophage reprogramming, and DC stimulation within a single platform, nanoparticles can directly coordinate the innate–adaptive interface [243,244,245]. This integrated approach has been shown to elevate costimulatory molecule expression, activate inflammatory transcriptional programs, and potentiate CD8^+^ T-cell infiltration into tumors. Hybrid nanoparticles that mimic both antigen-presenting cells and innate immune activators have demonstrated potential to enhance immune synapse formation. Incorporating these approaches into nanoparticles can amplify immune recognition of tumor antigens, upregulate immune activation-related genes, reduce immunosuppressive markers, and increase immunostimulatory proteins, thereby enhancing immune cell uptake and activation. These changes improve both immune cell uptake and downstream activation. Importantly, nanoparticles also enable combination therapeutic strategies by simultaneously engaging multiple arms of the tumor immunity cycle. For example, pairing nanoparticle-induced immune activation with therapies that promote ICD allows researchers to target distinct but complementary steps of the antitumor response. Such combinations not only enhance tumor-specific antigen release but also boost innate and adaptive immune mobilization, creating strong synergistic effects [246].

**Figure 7:**
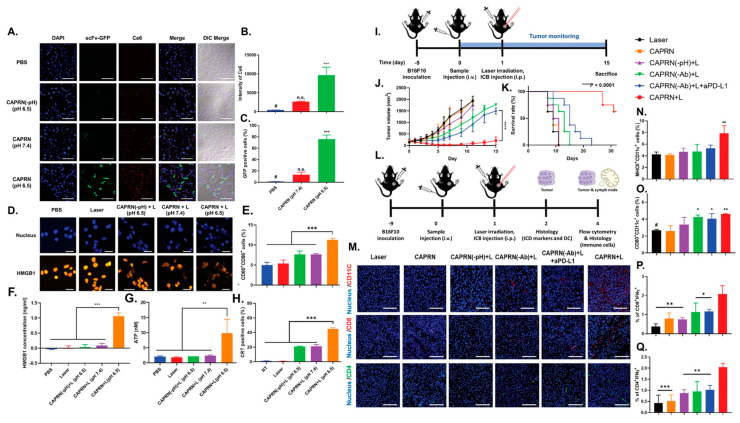
Cellular uptake and gene transfection of pH-responsive CAPRN in cancer cells. (**A**) Confocal microscopy image of B16F10 cells treated with CAPRN and CAPRN(-pH) under acidic or neutral conditions using fluorescence of intracellular Ce6 from CAPRN, green indicates fluorescence of expressed scFv-GFP, and blue corresponds to DAPI staining. The scale bar is 100 µm. (**B**,**C**) pH-responsive internalization of CAPRN and C scFv-GFP expression under acidic or neutral conditions, analyzed via flow cytometry. (**D**) Confocal microscopy image of (h) HMGB1 of B16F10 cells with various treatments. Scale bar is 20 µm. (**E**) In vitro DC maturation induced by supernatant of each group was analyzed via flow cytometry. In vivo therapeutic effects against B16F10-tumor-bearing mice. (**F**) HMGB1, (**G**) ATP in the supernatant measured using ELISA and ATP assay kit. (**H**) CRT-positive B16F10 cells analyzed via flow cytometry. (**I**) Overall experimental timeline showing treatment steps and procedures for evaluating therapeutic effects. (**J**) average tumor growth curve and (**K**) Kaplan–Meier survival curve of B16F10 bearing mice after different treatments. In vivo investigation of DC recruitment and activation, and T cell distribution. (**L**) Overall experimental timeline for the investigation of the immune response by various sample treatments. Immunofluorescence staining of CD11c, CD8, and CD4 (**M**) in the tumor site. Scale bar is 100 µm. (**N**,**O**) Flow cytometric quantitation of activated DCs (**N**), MHCII^+^ in CD11c, (**O**), CD80^+^ in CD11c in the tumor-draining lymph node. (**P**,**Q**) Flow cytometric quantification of activated CD8^+^ T cells (**P**), IFNγ^+^ in CD8^+^ activated T cells. (**Q**) IFNγ^+^ in CD4^+^ activated T cells. * *p* < 0.05; ** *p* < 0.01; *** *p* < 0.001; **** *p* < 0.0001; “ns”—no significant; (Reprinted with permission from Ref. [206]. Copyright 2025, John Wiley and Sons).

For example, Park et al. [206] were able to combine tumor-targeted antibody production and tumor-specific accumulation via pH-responsive and laser-assisted photodynamic leading to an immunologically active tumor (Figure 7). The design of CAPRN creates a multifunctional platform that allows tumor-specific accumulation via pH-responsive PEG detachment, targeted antibody release from dying tumor cells induced by laser-induced photodynamic therapy (PDT) and efficient intracellular gene delivery encoding anti-PD-L1 antibody. This nanoparticles was fabricated using Chlorin e6 (Ce6), a photosensitizer loaded into the pores of mesoporous silica nanoparticles (MSN), surface modification with NHS ester to enable subsequent chemical conjugation with a cationic polymer for the delivery of plasmid DNA (pDNA) and acid-cleavable imine linker can be dissociated under acidic conditions, leading to the detachment of PEG and exposing the cationic charge through residual amine groups on the nanoparticle surface. 

This design allowed for increased cellular uptake and gene expression of CAPRN under acidic conditions, shown by higher fluorescence intensity from both Ce6 and GFP expression profiles, suggesting that effective CAPRN internalization was induced by PEG detachment and led to effective expression of antibodies in response to an acidic environment. Combination with laser led to significant release of Immunogenic Cell Death (ICD) markers due to enhanced PDT effects. Use of the laser led to the highest levels of HMGB1, CRT, and ATP compared to the other groups. These induced ICDs also led to immune cell activation (DCs) under in vitro conditions. In vivo, combining laser irradiation and CAPRN injection exhibited the most robust tumor inhibition, shown through the highest survival rate and reduction of tumor size, further confirming the long-term antitumor efficacy. Further analysis showed CAPRN+L group exhibited a significant increase in the proportion of MHC II and CD80 positive DCs (CD11c-positive cells), which are established markers of DC maturation in the tumor. In addition to significantly improving the innate immune cell reservoir in the tumor, the immune response within the tumor and lymph nodes showed a rise in the number of lymphocytes (CD45-positive cells), highest helper CD4 (CD4^+^IFNr^+^) and effector CD8 (CD8^+^IFNr^+^) populations, providing evidence of the infiltration of activated T cells within the CAPRN+L groups. These results demonstrated that simultaneous activation of the immune cycle by ICD and ICB influences the DC population, leading to enhanced mature DCs migration to the tumor-draining lymph nodes (TDLNs) and activating T cells through physical interaction between DCs and T cells.

These platforms can promote DC–T cell crosstalk, enhance T-cell receptor (TCR) engagement, and increase the persistence of tumor-specific T cells within the TME. The combination of nanoparticle strategies that integrate multiple therapeutic mechanisms can greatly strengthen the antitumor immune response. By pairing treatments that induce immunogenic cell death (ICD) with approaches that activate and mobilize immune cells, researchers can target different stages of the tumor immunity cycle simultaneously. ICD promotes the release of tumor antigens and danger signals that activate innate immunity, while complementary immune-activating cues enhance adaptive responses, creating a synergistic effect. Incorporating both endogenous and exogenous vaccination elements further amplifies this process by boosting tumor antigen presentation and expanding tumor-specific cytotoxic T cells. Collectively, these multifaceted strategies optimize immune recognition, activation, and effector function, resulting in a more potent and durable antitumor response.

**Table 3:** Comparative Overview of Ligand-Decorated Nanoparticles Targeting TAMs, DCs, and Other Immune Cells.

Conjugated Nanoplatform	Ligand/Targeting Moiety	Receptor/Molecular Target	Target Immune Cell(s)	Selectivity/Response
Mannose-decorated PEG-*b*-AGE polymer-coated iron oxide nanoparticles	Mannose	Mannose receptor (MR)/CD206	M2-like TAMs	High M2 TAM uptake; repolarization to M1 [247]
Peptide-functionalized uIONP	M2pep	M2 TAM surface marker	M2-like TAMs	Selective M2 TAM targeting [248]
Folate-conjugated carboxymethyl chitosan nanoparticles carrying STAT3 siRNA	Folic acid	Folate receptor (FR)	M2-like TAMs	FR-mediated uptake in FR^+^ cells; STAT3 knockdown reduces expression, repolarizes TAMs from M2 to M1, inhibits tumor proliferation and enhances apoptosis in vitro and in vivo [109]
MAN-PLGA/ROS photogeneration nanoparticles	Mannose modification	Mannose receptor (CD206)	M2-like TAMs	Mannose-mediated uptake enriches NPs in M2 TAMs; light-triggered ROS reprograms TAMs to M1, enhances antigen presentation, activates T cells, and inhibits tumor growth and metastasis [249]
Anti-PD-L1-tethered lipid nanoparticle loaded with CSF1R inhibitor (α-PDL1-CSF-LNP)	Anti-PD-L1 monoclonal antibody on surface	PD-L1 on TAMs and tumor cells; CSF1R inside TAMs	M2-like TAMs	PD-L1–mediated binding enhances targeted delivery to PD-L1^+^ M2-like TAMs; CSF1R inhibitor reprograms TAMs toward M1 phenotype; increases phagocytic index and CD8^+^ T-cell infiltration [127]
PLGA nano-complexes encapsulating baicalin, CpG, and melanoma antigen Hgp peptide, surface-decorated with M2pep and α-peptide	M2pep+α-peptide	M2-like TAM surface markers	M2-like TAMs	M2pep/α-pep enhances uptake by M2-like TAMs; acidic lysosomal release of CpG and baicalin reverses M2-phenotype to M1, remodels the tumor microenvironment with increased inflammatory cytokines, boosts antigen presentation and T-cell activation, inhibits angiogenesis and tumor growth/metastasis [250]
Natural killer (NK) cell membrane-coated TCPP-loaded nanoparticles (NK-NPs)	NK cell membrane coating including innate receptors/proteins from NK cells	Tumor cell and TME components; engages tumor tissue and innate immune receptors (e.g., on macrophages)	TAM (promotes M1 polarization), NK cells	Biomimetic NK membrane enables tumor targeting and immune modulation; photosensitizer TCPP enables photodynamic tumor cell killing; NK-NPs induce M1 polarization of TAMs, enhance antitumor immunity, inhibit primary tumor growth, and produce abscopal (distant) tumor suppression [251]
Dual-targeting peptide-decorated nanoparticles (M2NPs) delivering anti-CSF-1R siRNA	Fusion peptide combining α-peptide (SR-B1 targeting) and M2pep (M2 macrophage binding)	Scavenger receptor B type 1 (SR-B1) and M2 macrophage surface target; CSF-1R for siRNA silencing	M2-like TAMs	Dual targeting enhances nanoparticle uptake specifically by M2-like TAMs vs. tissue macrophages; anti-CSF-1R siRNA depletes immunosuppressive TAMs, reduces IL-10 and TGF-β, increases IL-12/IFN-γ and CD8^+^ T-cell infiltration, restores T-cell function and suppresses melanoma growth in vivo [252]
Albumin-based biomimetic nanoparticles (T12/Man-BSA NPs) codelivering disulfiram/copper complex and regorafenib	Transferrin receptor-binding peptide T12 and mannose	Transferrin receptor (TfR) and secreted protein acidic and rich in cysteine (SPARC) for BBB and glioma; SPARC and mannose receptor (MR) on M2 TAMs	M2-like TAMs; T cells (indirect activation)	Dual targeting enables BBB penetration and glioma delivery; reprograms TAM M2 to M1, remodels immunosuppressive TME, enhances CD8^+^ T-cell infiltration, and synergizes chemotherapy with macrophage-directed immunotherapy to suppress glioma growth [253]
SA/IBR/EPG nanocomplexes: sialic acid–stearic acid conjugate modified egg phosphatidylglycerol nanoparticles encapsulating ibrutinib	Sialic acid–stearic acid conjugate (SA) on nanocomplex surface; Bruton’s tyrosine kinase (BTK) inside TAMs	Sialic acid receptors/Siglec-expressing TAMs (overexpressed on tumor-associated macrophages)	M2-like TAMs	SA-mediated uptake enhances delivery to TAMs; inhibits BTK in TAMs; reduces angiogenesis and tumorigenic cytokine release; suppresses tumor progression with preferential TAM accumulation and low systemic side effects [254]
Photosensitizer-loaded upconversion nanocrystals with MnO_2_ nanosheets and hyaluronic acid (HA-UCN-MnO_2_)	Hyaluronic acid (HA) surface and MnO_2_ integrated scaffold	HA receptor (CD44) on tumor cells & TAMs; oxygen generation via MnO_2_ reacting with H_2_O_2_	M2-like TAMs	NIR (808 nm)-activated PDT enhanced by MnO_2_-mediated oxygen generation alleviates tumor hypoxia; HA engages TAMs and tumor cells, reprograms M2 TAMs toward pro-inflammatory M1 phenotype, enhancing PDT efficacy and inhibiting tumor recurrence by remodeling hypoxic TME and immune microenvironment [255]
Dual nanocarrier system: TAM-targeting liposome (AAN-Lip-Regorafenib)+cancer-cell-penetrating polymer (iRGD-HD)	Alanine-alanine-asparagine (AAN) for TAMs; iRGD peptide for tumor cells	M2-like TAM localization at perivascular niche; integrin receptors (iRGD-mediated) on tumor cells	M2-like TAMs	AAN-Lip-Regorafenib accumulates in TAM-rich perivascular areas and repolarizes TAMs from M2 to M1, reducing immunosuppression; iRGD-HD penetrates deep tumor tissue, induces immunogenic cell death, enhances CD8^+^ T-cell infiltration, and jointly suppresses metastatic tumor growth [256]
Dual-responsive polypeptide nanovectors (sPEG/GLC/miR155	Mannose	Mannose receptor (CD206)	M2-like TAMs; T cells and NK cells (indirect activation)	Mannose-mediated uptake enriches miR155 delivery in TAMs; dual pH/redox responsiveness promotes release in the tumor microenvironment; miR155 reprograms TAMs to M1 phenotype, increases pro-inflammatory markers, and enhances T-cell/NK activation leading to robust tumor inhibition in vivo [164]
Baicalin-loaded PLGA nanoparticles with antigenic peptide and CpG, coated with galactose-inserted erythrocyte membrane	Galactose-inserted erythrocyte membrane coating	Mannose/galactose receptors on TAMs and antigen presentation pathways	M2-like TAMs and T cells (indirect activation)	Biomimetic coating enhances uptake by TAMs; baicalin and CpG polarize M2 TAMs to M1 phenotype, increase CD4^+^/CD8^+^ T-cell infiltration, improve immune activation, and suppress melanoma growth in vivo [257]
PLGA nanoparticles encapsulating ovalbumin+TLR3/TLR7 ligands, surface-decorated with monoclonal antibodies against CD40, DEC-205 or CD11c	Monoclonal antibodies (anti-CD40, anti-DEC-205, anti-CD11c) coupled to particle surface	CD40 (TNF-α family receptor), DEC-205 (C-type lectin), CD11c (integrin) on dendritic cells (DCs)	Dendritic cells	Antibody-mediated targeting enhances NP uptake by DCs compared with non-targeted NP; all three receptor-targeted NPs similarly increase DC activation (IL-12, co-stimulatory markers) and prime potent CD8^+^ T-cell responses with strong proliferation and IFN-γ production in vitro and in vivo compared to untargeted NP controls [258]
Lipid-coated calcium phosphate nanoparticles (LCP NPs) encapsulating modified BRAF^V600E peptide and CpG ODN adjuvant	Mannose-modified PEGylated LCP NP delivering peptide antigen+CpG	Mannose receptor (enhanced DC targeting); antigen presentation via MHC I on DCs	Dendritic cells; indirect effects on macrophages in TME	Nanoparticle-mediated delivery promotes efficient DC uptake and maturation, elicits robust antigen-specific CD8^+^ T-cell responses, remodels immunosuppressive TME (increased CTL infiltration, enhanced M1/M2 ratio), and inhibits tumor growth in BRAF-mutant melanoma model [259]
PLGA nanoparticles encapsulating melanoma antigen peptide (hgp100_25–33_) and MPLA adjuvant, coated with erythrocyte membrane	Erythrocyte membrane-coated nanoparticle with mannose conjugation for dendritic cell targeting	Mannose receptor on antigen-presenting cells (APCs); antigen presentation via MHC I/II	Dendritic cells; antigen-specific CD8^+^ T cells	Biomimetic erythrocyte membrane enhances circulation and immune evasion; mannose conjugation promotes uptake by APCs in lymphoid organs; nanoparticle vaccine increases IFN-γ secretion, enhances CD8^+^ T-cell response, delays tumor onset, and inhibits melanoma growth and metastasis [260]
Fe_3_O_4_ nanoparticles covalently bound to ovalbumin antigen (Fe_3_O_4_-OVA)	Covalent conjugation of ovalbumin (OVA) antigen	Antigen presentation receptors (MHC) and pattern recognition receptors on APCs	Dendritic cells; macrophages (indirect activation); T cells (indirect activation via antigen presentation)	Protects antigen and enhances uptake by dendritic cells, promotes DC maturation and T-cell priming, indirectly stimulates macrophages via cytokine signaling; inhibits primary tumor growth and prevents lung metastasis of melanoma in vivo [261]
Antigen-capturing nanoparticles (AC-NPs) formulated from PLGA or surface-modified polymers that bind tumor-derived proteins	Surface chemistries (e.g., PLGA, NH_2_-PEG, DOTAP, maleimide-PEG) enabling tumor antigen capture	Tumor-derived protein antigens captured post-radiotherapy; presented via MHC on APCs	Dendritic cells	Captures tumor antigens released after radiotherapy and delivers them to APCs, enhances antigen presentation, increases CD8^+^ cytotoxic T-cell expansion and CD4^+^/CD8^+^ ratios relative to Treg, synergizes with αPD-1 immunotherapy and improves the abscopal effect in melanoma models [262]
Liposomes-coated gold nanocages (Lipos-AuNCs) loaded with melanoma antigen peptide TRP2 and adjuvant MPLA, surface-modified with anti-CD11c antibody	Anti-CD11c monoclonal antibody on liposome coating	CD11c on dendritic cells (DCs); antigen presentation via MHC I/II	Dendritic cells; T cells (indirect activation via antigen presentation)	CD11c targeting enhances uptake by DCs, promotes DC activation and maturation, increases antigen presentation to T cells, stimulates robust CD8^+^ T-cell responses, and inhibits tumor growth and metastasis in melanoma models with in vivo tracking capability [263]
PLGA nanoparticles co-loaded with STING agonist and tumor antigens, coated with engineered peptide-expressed biomimetic cancer cell membrane (PLGA/STING@EPBM)	Engineered peptide-expressed biomimetic cancer cell membrane (EPBM) facilitating targeting	Clec9a receptor on Clec9a^+^ dendritic cells; STING activation pathway	Dendritic cells	Biomimetic coating promotes NP uptake by Clec9a^+^ DCs; enhanced IFN-I gene expression and antigen cross-presentation; robust antitumor immunity; improved efficacy of STING agonist with synergistic effect with radiotherapy [264]
Liposomal Fc-conjugated cancer vaccine	Fc domain covalently attached to liposomal cancer vaccine	Fc receptors (FcγRs) on antigen-presenting cells (DCs, macrophages) and B cells	Dendritic cells; macrophages; B cells; T cells (indirect activation via antigen presentation)	Fc domain enables engagement with Fcγ receptors, enhancing uptake by APCs, boosting both humoral (antibody) and cellular (T-cell) immune responses compared with non-Fc liposomal vaccines [265]
Dextran derivative-modified pH-sensitive liposomes loaded with ovalbumin antigen	pH-responsive 3-methylglutarylated dextran (MGlu-Dex) on liposome surface	Endosomal pH (acidic environment) and antigen processing machinery; uptake by antigen-presenting cells	Dendritic cells; T cells (indirect activation)	pH-responsive MGlu-Dex enables endosomal escape and cytosolic delivery of antigen to DCs, enhancing antigen cross-presentation and induction of both humoral and cellular antitumor immunity; suppresses tumor growth and prolongs survival in EG7-OVA tumor model [266]
ASPIRE nanovaccine—cell membrane nanovesicles derived from recombinant adenovirus-infected dendritic cells	pMHC-I (tumor antigen peptide–MHC I complexes), anti-PD-1 antibody, B7 co-stimulatory molecules all anchored on the engineered DC membrane	pMHC-I engages T-cell receptors; PD-1/PD-L1 pathway blockade; B7/CD28 co-stimulation enhances T-cell activation	Dendritic cells (source), T cells (naive and exhausted)	Biomimetic DC-derived vesicles present antigen directly to T cells (“antigen self-presentation”), reverse immunosuppression via anti-PD-1, improve lymph node targeting, generate broad-spectrum T-cell responses that eliminate established tumors and enhance personalized cancer immunotherapy [267]
Liposome vaccine co-loading palmitoylated synthetic long peptides (SLP) and α-galactosylceramide (αGC), with optional palmitoylated Le^γ^ glycan	SLP antigen; α-galactosylceramide; palmitoylated Lewis Y (Le^γ^) glycan for targeting	C-type lectin receptors, such as DC-SIGN and Langerin on dendritic cells; CD1d for αGC presentation	Dendritic cells; CD8^+^ T cells (indirect activation); invariant NKT cells (iNKT)	Targeted uptake by DC subsets via DC-SIGN/Langerin enhances antigen and αGC presentation; induces robust tumor-specific CD8^+^ T-cell responses and iNKT activation with strong IFN-γ secretion, supporting both adaptive and innate antitumor immunity [268]

**Table 4:** Nanomaterials Co-Targeting TAMs and DCs for Synergistic Cancer Immunotherapy.

Conjugated Nanoplatform	Ligand/Targeting Moiety	Receptor/Molecular Target	Selectivity/Response
Core–shell nanoscale coordination polymer (PPA/HG) with TLR3 agonist core and cholesterol-conjugated prodrug shell	Polyinosinic:polycytidylic acid (PPA) in core; cholesterol-conjugated 3-(hydroxyolinoyl)glycine (HG) on shell	TLR3 on phagocytes (DCs, TAMs); KDM3A/c-Myc pathway regulating CD47/PD-L1 in cancer cells	TLR3 activation in DCs and TAMs promotes DC maturation and macrophage repolarization to M1 phenotype; HG downregulates CD47 and PD-L1 expression in tumor cells, enhancing phagocytosis and CTL infiltration while decreasing regulatory T cells; potent inhibition of tumor progression in TNBC and PDAC models with low toxicity [205]
HA-DOX/PHIS/R848 nanoparticles: hyaluronic acid–doxorubicin prodrug-coated pH-responsive poly(L-histidine) core co-delivering immunomodulator R848 and chemotherapeutic DOX	Hyaluronic acid (HA) as targeting moiety on nanoparticle surface	CD44 receptor on breast cancer cells	HA mediates CD44-dependent uptake by breast cancer cells; pH sensitivity enables spatially controlled release of R848 (immune activation) and DOX (cytotoxicity); enhances tumor-targeting, immune modulation, and synergistic suppression of 4T1 breast tumors in vivo by combining immunotherapeutic and chemotherapeutic effects [269]
Surface-engineered tumor-derived antigenic microparticle vaccine (Fe_3_O_4_/T-MPs-CpG/Lipo)	Tumor-derived antigenic microparticles with CpG-loaded liposome arrays	Antigen presentation receptors on APCs (e.g., TLR9), TAM (modulated by Fe_3_O_4_)	CpG promotes APC activation; Fe_3_O_4_ reprograms TAMs from M2 toward M1 phenotype; enhances tumor antigen presentation, increases cytotoxic T-cell infiltration, converts “cold” tumor to “hot,” synergizes with PD-L1 blockade to suppress tumor growth and extend survival [270]
Mannose-decorated polydopamine-coated PLGA nanoparticles loading R848 (Man-pD-PLGA-NP@R848)	Surface-decorated mannose	Mannose receptor (CD206) on TAMs and tumor-infiltrating DCs; TLR7/8 (activated by R848)	Mannose receptor–mediated uptake enhances delivery to TAMs and TIDCs; R848 activates TLR7/8, reprogramming TAMs toward pro-inflammatory (M1) phenotypes, increases CD4^+^/CD8^+^ T-cell infiltration, decreases angiogenesis, and converts “cold” to “hot” TME, suppressing tumor growth in vivo [271]
Mannose-modified cationic polymer/let-7b nanocomplexes	Mannose	Mannose receptor (CD206) on TAMs and tumor-infiltrating DCs	Mannose-mediated uptake enriches let-7b in TAMs and TIDCs; delivered let-7b activates TLR7 and inhibits IL-10, reprogramming immunosuppressive cells to pro-inflammatory phenotypes and reversing the suppressive TME, leading to tumor rejection in vivo [272]
Core–shell nanoscale coordination polymer (PPA/HG) co-delivering TLR3 agonist (polyinosinic–polycytidylic acid, PPA) and cholesterol-conjugated HG prodrug	None (TLR3 agonist activates APCs; HG prodrug modulates tumor checkpoints)	TLR3 on APCs (DCs, TAMs); CD47 and PD-L1 on tumor cells	TLR3 activation matures DCs and repolarizes TAMs to M1; HG downregulates CD47/PD-L1 in tumor cells, promoting phagocytosis and T-cell cytotoxicity; synergistic innate and adaptive antitumor responses with tumor regression and low toxicity [205]
ROS-responsive HSA-based PEG/IL12-IA nanoparticles co-loading indocyanine green (ICG), arginine (Arg), and IL-12	None (ROS-responsive release; passive accumulation)	ROS-responsive nanoparticle releases IL-12 in the tumor, activating immune cells.	ROS-triggered IL-12 release reprograms M2 TAMs to M1; phototherapy induces immunogenic cell death, activating DCs and T cells; synergistically enhances antitumor immunity and suppresses tumor growth/metastasis [196]
pIL-12 + PLX3397 encapsulated in cRGD-functionalized PssPD nanoparticles	cRGD peptide	αvβ3 integrins on tumor cells/tumor vasculature	cRGD enhances tumor accumulation; PLX3397 inhibits CSF1R to deplete/reprogram M2 TAMs; IL-12 polarizes TAMs to M1 and activates DCs and T cells; synergistic tumor inhibition and immune activation [61]
Hf DBP MOF	TLR 7 agonist (Imiquimod, IMD) + Anti CD47 antibody	TLR7 + CD47	The combination of treatments led to increased antitumor immunity in vivo. Hf-MOF sensitizes tumors to radiotherapy, inducing immunogenic cell death IMD and αCD47 promote M2-M1 TAM polarization, dendritic cell maturation, and T-cell activation [191]

## 6. Biomimetic and Stimuli-Responsive Nanocarriers for Precision Tumor Targeting and Immune Modulation

Nano-enabled modulation of TAMs and DCs offers an enhanced ability to selectively target cancerous tissues while reducing the systemic toxicities associated with conventional chemotherapy. However, their therapeutic potential is often restricted by factors such as poor biocompatibility with the body’s immune system [273] and lack of stimuli-responsive controlled drug release [274]. This section highlights recent advances in biomimetic and stimuli-responsive nanocarriers designed to overcome these challenges, alongside a critical discussion of translational considerations.

A major barrier to effective NP-based therapy is rapid immune recognition. Many NPs are quickly identified as “nonself” by the body, leading them to be quickly expelled by the immune system without a chance to accumulate at the tumor site. To overcome this, two primary classes of biomimetic carriers, cell membranes and extracellular vesicles (EVs), have been engineered to cloak NPs and improve in vivo persistence [273]. For example, Blaya-Cánovas et al. [275] developed an exhausted T-cell (NExT) membrane-camouflaged PLGA NP platform to exploit tumor immune checkpoint ligand expression for targeted delivery. Tumors express ligands such as PD-L1, Galectin-3, FGL-1, MHCII, Galectin-9, HMGB1, and CEACAM-1, which bind to T-cell checkpoint receptors (PD-1, LAG-3, TIM-3) and drive T-cell exhaustion. By coating NPs with NExT membranes, these ligand–receptor interactions are repurposed for tumor targeting. Using TNBC cell line SUM159 and additional breast cancer lines (MCF7, MDA-MB-231, MDA-MB-468, BT549, and Hs578T), they first quantified ligand expression and confirmed higher PD-L1 levels in TNBC. In vitro studies demonstrated markedly enhanced and accelerated NP accumulation with NExT carriers. In CD1 mice bearing patient-derived TNBC xenografts, NExT-coated NPs showed improved intratumoral retention and chemotherapeutic efficacy. Other biomimetic strategies include cloaking NPs with membranes from red blood cells, white blood cells, and platelets to enhance circulation and targeting [273]. Deng et al. [276] developed genetically engineered T-cell–membrane-camouflaged nanoparticles that were designed to improve tumor homing and activate immunity in bladder cancer. The membrane coating preserved CD3ζ- and CXCR3-mediated targeting, increasing intratumoral accumulation by ~2–3-fold. Loaded copper ions induced cuproptosis, resulting in >60% tumor-cell death in vitro, while the photothermal core achieved a ΔT ≈ of 20–25 °C under NIR irradiation. In mice, the combined cuproptosis–photothermal therapy reduced tumor volumes by ~80–90%, elevated CD8^+^ T-cell infiltration, and significantly prolonged survival compared with monotherapies.

**Figure 8:**
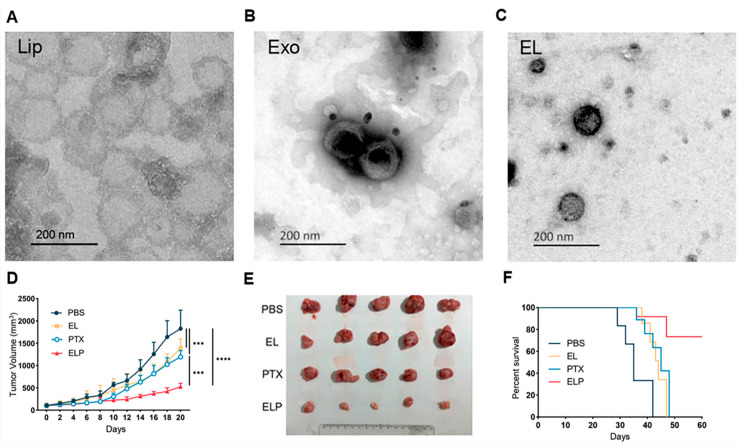
Engineered paclitaxel-loaded hybrid exosomes enhance tumor-targeted drug delivery and improve anticancer efficacy. (**A**–**C**) Transmission electron microscopy morphologies of the liposomes, exposomes, and hybrid exosomes (EL), respectively. (**D**) Tumor growth curves, (**E**) excised tumor sizes at day 16, and (**F**) percent survival) of CT26-bearing mice. *** *p* < 0.001, **** *p* < 0.0001; (Reprinted with permission from Ref. [274]. Copyright 2024, Yan Chen et al.).

Exosome-based carriers have also been explored. Li et al. [277] developed a bioinspired exosome-liposome hybrid nanoparticle to co-deliver triptolide (TP) and miR497, aiming to overcome chemoresistance in ovarian cancer. The hybrid nanoparticles combined tumor-derived exosomes, which enhance cellular uptake and immune evasion, with cRGD-modified liposomes for tumor targeting. In cisplatin-resistant ovarian cancer cell lines, the codelivery system significantly increased cellular uptake, induced apoptosis, and synergistically suppressed the PI3K/AKT/mTOR pathway. In vivo, these nanoparticles preferentially accumulated in tumors, inhibited tumor growth, promoted ROS generation, and repolarized TAMs from M2 to M1 phenotype, all with minimal systemic toxicity in mice. Similarly, Jiménez-López et al. [278] utilized magnetoliposomes, combining liposomes and superparamagnetic iron oxide nanoparticles (SPIONs), enabling magnetic hyperthermia-triggered drug release and immune modulation by promoting immunogenic cell death in tumor cells. The localized hyperthermia induced by the magnetic field can enhance the release of tumor-associated antigens and damage-associated molecular patterns (DAMPs), stimulating dendritic cells and cytotoxic T-cell responses. This dual function highlights the potential of magnetoliposomes as both therapeutic and immune-activating nanocarriers. Hybrid systems combining synthetic and natural carriers have further improved efficacy. Wang et al. [274] developed a hybrid exosome–liposome (EL) system combining mesenchymal stem cell-derived exosomes with folate-modified synthetic liposomes to enhance chemotherapy delivery to breast cancer (Figure 8). Synthetic liposomes provide high drug-loading capacity and consistent production, while exosomes offer biomimetic immune evasion. Paclitaxel (PTX) loaded in the hybrid (ELP) activated CD4^+^/CD8^+^ T cells, reduced M2 TAMs, promoted M1 polarization, and decreased regulatory T cells. Exosomes were confirmed via CD9, TSG101, and CD63 markers. In BALB/c mice bearing CT26 tumors, ELP showed superior tumor uptake, resulting in enhanced macrophage repolarization, T-cell activation, and overall chemotherapy efficacy compared with PTX or EL alone.

Plant-derived vesicles (PDVs) represent an emerging and versatile class of biomimetic nanocarriers with unique advantages for cancer therapy. Unlike conventional synthetic NPs or mammalian EVs, PDVs are naturally biocompatible, non-immunogenic, and can be produced at scale from renewable plant sources, offering a cost-effective and sustainable platform. Corvigno et al. [279] developed a hybrid system combining watermelon-derived PDVs with a third-generation PAMAM dendrimer loaded with miRNA146 mimic to enhance RNA delivery to tumor cells. The PDVs facilitated efficient cellular uptake, protected the miRNA payload from degradation, and promoted selective accumulation in tumor tissues. In vitro studies demonstrated potent anti-angiogenic effects, inhibition of tumor cell proliferation, and modulation of key signaling pathways associated with tumor growth. In vivo experiments in mouse tumor models confirmed significant tumor suppression, primarily through remodeling of the TME and activation of anti-tumor immune responses, including enhanced infiltration of cytotoxic T cells and polarization of TAMs toward the M1 phenotype. Despite these advances, clinical translation of cell membrane and EV-based carriers remains limited by production scalability, cost, and batch-to-batch variability, which can affect biocompatibility, functional consistency, reproducibility, and incomplete understanding of transport mechanisms [273]. Artificial intelligence (AI) driven predictive models may help improve NP stability and manufacturing consistency [280].

Beyond targeting, personalization of NPs based on an individual’s genetic and molecular profile enhances therapeutic precision. Ashkarran et al. [281] demonstrated sex-dependent differences in NP protein coronas, reflecting plasma metabolite and protein variations that influence NP circulation, immune recognition, and tumor targeting. These findings underscore that biological context, including sex, metabolic state, and individual plasma composition, can significantly alter NP behavior, highlighting the necessity of personalized NP design. While challenges in manufacturing consistency, systemic toxicity, biocompatibility, and structural stability continue to limit fully personalized NPs [280], combinatorial NP-mediated chemotherapy offers a practical approach to individualized treatment. Integrating molecular profiling with hybrid NP platforms can help mitigate variability and improve translational potential. For example, Bar-Natan et al. [282] tested 17 kinase inhibitors across four osteosarcoma cell lines (U2OS, MG-63, SaOS-2, K7M2) to assess cell-specific sensitivity. Using SynergyFinder, the researchers identified optimal drug combinations, which were delivered in alternating schedules via NPs in K7M2 murine models. This regimen not only enhanced tumor cell apoptosis but also minimized off-target toxicity in major organs. The study demonstrated that integrating molecular profiling, combinatorial drug selection, and NP delivery could tailor cancer therapy to specific tumor characteristics, providing a proof-of-concept for precision nanomedicine that accounts for inter-patient variability. 

**Figure 9:**
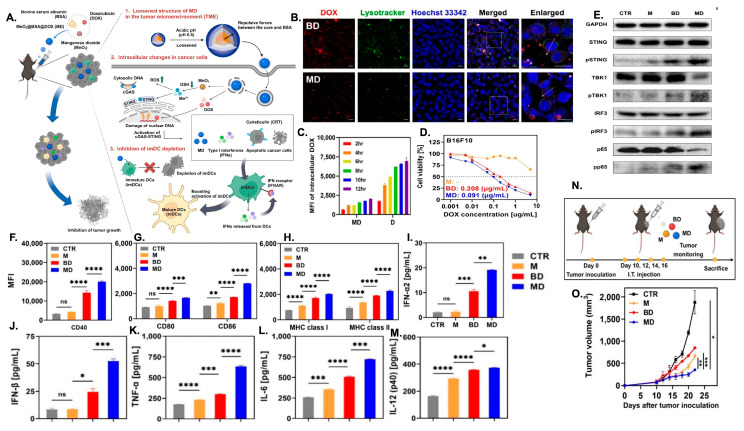
pH- and redox dual-responsive MnO_2_@BSA@DOX nanoparticle for tumor-selective intracellular drug release and activates the cGAS–STING signaling pathway, thereby promoting dendritic cell maturation and enhancing antitumor immune responses. (**A**) Schematic illustration of internalization and intracellular changes in selective cytotoxicities and the cGAS-STING pathway activation by MD against B16F10 cells. (**B**) CLSM images of lysosomal escapes of BD and MD. Scale bar: 20 μm. (**C**) Cellular uptake of free DOX and MD (*n* = 3). (**D**) Evaluation of in vitro cytotoxicity of MD in B16F10 cells. (**E**) cGAS-STING pathway activation. (pSTING/STING, pTBK1/TBK1, pIRF3/IRF3, and pp65/p65). Enhanced DC maturation. Expression levels of (**F**) CD40, (**G**) CD80/CD86, and (**H**) MHC class I/MHC class II. (**I**–**M**) Quantification of (**I**) IFN-α2, (**J**) IFN-β, (**K**) TNF-α, (**L**) IL-6, and (**M**) IL-12 (p40) released from B16F10 cells and DCs. (**N**) Schematic illustration of intratumoral administration of M, BD, and MD in the B16F10 murine melanoma model. (**O**) Average tumor growth curves. * *p* < 0.05; ** *p* < 0.01; *** *p* < 0.001; **** *p* < 0.0001; “ns”—no significant; (Reprinted with permission from Ref. [284]. Copyright 2024, American Chemical Society).

Aside from reaching the tumor site, a critical function of NP-based cancer therapy is the controlled release of therapeutics. Stimuli-responsive crosslinked nanomedicines (SCNs) offer precise control over drug delivery, utilizing chemical bonds that respond reversibly to tumor-specific triggers such as pH, enzymes, redox conditions, or ROS. These triggers can induce changes in NP size, charge, and integrity, enabling spatially and temporally controlled anti-tumor drug release [283]. For instance, Yun et al. [284] designed a pH-and redox-dual-responsive MnO_2_@BSA@DOX nanoparticle (MD) that exemplifies how stimuli-responsive nanocarriers can integrate tumor targeting with immune modulation. The nanoparticle releases DOX selectively in the acidic tumor microenvironment while MnO_2_ is reduced to Mn^2+^, generating ROS and activating the cGAS–STING pathway. This leads to type I interferon production, calreticulin exposure on tumor cells, and enhanced DC maturation (CD40^+^, CD80^+^, CD86^+^) along with IL-12 secretion. These changes facilitate effective T-cell priming and amplify antitumor immunity (Figure 9). In murine melanoma models, MD suppressed tumor growth, extended survival, and induced immunogenic tumor cell death without systemic toxicity. Sun et al. [285] designed tumor-targeting, redox-responsive, photo-crosslinked nanogels (TRNGs) from hyaluronic acid (HA) conjugated with cytochrome C (CC), which disassemble under elevated glutathione (GSH) levels characteristic of tumor cells. HA facilitated CD44-mediated targeting, resulting in higher uptake by A549 (CD44+) cells compared to HepG2 (CD44−). In vivo, TRNGs achieved maximal tumor accumulation, reduced tumor size, and minimal systemic toxicity. Going beyond single-stimulus systems, recent work has shown dual-stimuli (pH/ROS) responsive nanogels for combined chemo-photodynamic therapy. Yuan et al. [286] co-encapsulated doxorubicin (DOX) and a near-infrared (NIR) photosensitizer (ICG-BSA) in a pH/ROS dual-sensitive nanogel. Under the acidic and oxidative tumor microenvironment, PEG shielding was removed to expose cell-penetrating peptides and enable cellular uptake. Subsequently, NIR irradiation generated ROS, triggering nanogel disintegration and DOX release, resulting in synergistic antitumor effects and enhanced tumor-selective cytotoxicity. Similarly, Zhou et al. [287] developed a pH/enzyme-responsive inorganic–organic hybrid nanomedicine. For instance, a zinc oxide–based nanoplatform coated with an enzyme-sensitive polymer exhibited Matrix Metalloproteinase-2 (MMP-2) enzyme and acidic pH–pH-triggered DOX release, leading to effective drug delivery, enhanced penetration in multidrug-resistant (MDR) tumor models, and improved in vivo antitumor efficacy.

Emerging strategies, such as anti-phagocytosis-blocking, repolarization-resistant, membrane-fusogenic liposomes (ARMFUL), enable one-step multiplexed cell engineering. ARMFUL delivers anti-CD47 on the surface and BLZ945 in the cytoplasm, promoting macrophage phagocytosis, maintaining M1 polarization, enhancing antigen presentation, and inducing T-cell activation. This multifunctional approach demonstrates potent anti-tumor immunity with reduced off-target effects [288]. Integrating these approaches with biomimetic, stimuli-responsive, and personalized designs maximizes tumor-specific delivery, controlled drug release, and immune modulation while minimizing off-target toxicity. Collectively, these innovations lay the groundwork for next-generation precision nanomedicine in cancer immunotherapy. Biomimetic and stimuli-responsive nanocarriers offer significant advantages for precise tumor targeting, immune modulation, and controlled drug release. Hybrid systems and personalized NP design further enhance therapeutic efficacy while minimizing systemic toxicity. However, clinical translation remains limited by challenges in large-scale production, batch-to-batch variability, and reproducibility, which must be addressed to ensure consistent performance and regulatory approval. Future research should focus on scalable manufacturing methods, standardization of carrier sources, and AI-guided optimization to bridge the gap between preclinical promise and clinical application.

## 7. Adaptive Resistance Mechanisms in Nano-Immunotherapy

Despite the substantial promise of nano-immunotherapy in enhancing immune cell infiltration, improving delivery of immunomodulators, and remodeling the tumor microenvironment (TME), tumors can still develop adaptive resistance mechanisms that limit the long-term therapeutic efficacy of even nanoparticle-enabled strategies. One well-characterized mode of resistance is the upregulation of alternative immune checkpoints following PD-1/PD-L1 blockade. Studies in multiple cancer models, including preclinical mouse models and clinical samples, have documented compensatory increases in inhibitory receptors such as T cell immunoglobulin and mucin domain 3 (TIM-3), Lymphocyte-activation gene 3 (LAG-3), T cell immunoreceptor with Ig and ITIM domains (TIGIT), and V-domain Ig suppressor of T cell activation (VISTA) which sustain T-cell exhaustion and immune suppression when PD-1 pathways are inhibited [289,290,291]. Importantly, in the context of nanoparticle-enhanced immunotherapy, preclinical work with the hafnium oxide nanoparticle NBTXR3 combined with PD-1 blockade in anti-PD-1-resistant lung cancer models demonstrated that this combination significantly upregulated LAG-3 and TIGIT expression, and that dual blockade of these checkpoints was required to achieve superior antitumor outcomes, providing direct evidence that adaptive immune resistance can persist even with nanoparticle-enabled therapy [292,293].

Beyond checkpoint compensation, metabolic adaptation within the TME, including hypoxia, nutrient depletion, and accumulation of immunosuppressive metabolites such as lactate, can suppress effector T-cell function, skew macrophages toward immunosuppressive phenotypes, and impair dendritic cell (DC) maturation and antigen presentation. Nanoparticle-based strategies targeting hypoxia or tumor metabolism can partially restore immune activity, but the dynamic and heterogeneous metabolic landscape may still allow adaptive immune escape [294,295]. Furthermore, the functional plasticity of TAMs and DCs can limit the durability of nano-immunotherapy. Nanoparticle-mediated strategies can transiently reprogram TAMs toward a pro-inflammatory M1-like phenotype and enhance DC maturation. However, in the immunosuppressive TME, TAMs may revert to M2-like states, and DCs can become tolerogenic or dysfunctional, reducing the persistence of antitumor immunity [72,296]. Collectively, these mechanisms underscore that potential tumor resistance remains a critical consideration in nano-immunotherapy, emphasizing the need for rational multi-targeted and combinatorial designs to achieve durable clinical responses.

## 8. Challenges, Future Perspectives, and Conclusions

Nano-enabled modulation of TAMs and DCs has emerged as a highly promising strategy to overcome immunosuppressive barriers within the TME and enhance cancer immunotherapy. However, several critical challenges must be addressed to enable safe and effective clinical translation. A major concern is off-target immune activation and immunotoxicity. Nanoparticles delivering potent immunomodulators, such as toll-like receptor agonists, STING agonists, cytokines, or nucleic acids, may inadvertently trigger systemic inflammation, cytokine release syndrome, or autoimmunity. While clinically approved immune checkpoint inhibitors (ICIs), including anti-PD-1 (nivolumab, pembrolizumab) and anti-CTLA-4 (ipilimumab), have demonstrated durable responses, their combination with nano-immunomodulators could exacerbate immune-related adverse events, necessitating highly selective TAM/DC targeting, biomimetic coatings, and stimuli-responsive payload release.

Tumor heterogeneity and inter-patient variability further complicate translation. The density and polarization state of TAMs, the abundance and maturation of DCs, and the broader TME landscape vary across cancer types, stages, and individual patients, influencing nanoparticle uptake, immune engagement, and therapeutic efficacy. Personalized strategies guided by immune profiling, molecular biomarkers, and imaging techniques are therefore critical to optimize outcomes.

Scalability, reproducibility, and regulatory challenges also pose significant hurdles. Multifunctional nanomaterials often involve complex synthesis, multi-component payloads, and surface functionalization, which can lead to batch-to-batch variability. Ensuring consistent physicochemical properties, stability, and immunological performance is essential for regulatory approval. Integration with clinically approved therapies, including ICIs, CAR-T therapy, or cytokine-based treatments (e.g., IL-2 analogs) requires careful assessment of synergistic efficacy and safety profiles. Interdisciplinary approaches combining advanced preclinical models, standardized manufacturing protocols, and early regulatory engagement will be essential to accelerate translation.

Looking forward, emerging strategies promise to overcome these challenges. Biomimetic nanocarriers, such as cell membrane-coated nanoparticles and exosome-inspired vesicles, improve immune targeting, circulation time, and biocompatibility. Stimuli-responsive and spatiotemporally controlled systems enable precise TAM/DC modulation within the TME, minimizing systemic toxicity. The integration of nano-enabled TAM/DC modulation with multimodal immunotherapies, including ICIs, adoptive cell transfer, cancer vaccines, and radiotherapy, can potentiate antigen presentation, T-cell recruitment, and immunogenic tumor cell death. Personalized nanomedicine approaches, guided by patient-specific immune profiling, can further address tumor heterogeneity and optimize therapeutic efficacy.

In conclusion, nano-enabled reprogramming of TAMs and DCs represents a versatile and potent avenue to remodel the TME, amplify adaptive immune responses, and enhance the efficacy of existing cancer immunotherapies. By combining targeted immune modulation with multimodal and personalized strategies, these platforms have the potential to convert immunologically “cold” tumors into “hot,” responsive ones. Addressing immunotoxicity, tumor variability, and translational barriers will be pivotal in realizing the clinical impact of these approaches. Continued innovation in nanomaterial design, combinatorial therapies, and regulatory frameworks will drive the next generation of precision immunotherapies, offering broader applicability, improved safety, and durable antitumor responses.

## Figures and Tables

**Table 1:** RNA nanotherapeutics for remodeling TAMs.

RNA Types	Nanocarrier	Targeted Gene/Pathway	Key Findings
miR-125b	Dual CD44/EGFR-targeted hyaluronic acid (HA) based polymeric nanoparticles	Delivery of miR-125b/wt-p53 transfection	Macrophage repolarization, downregulation of antiapoptotic gene (e.g., Bcl-2) [161]
miR-125b	HA-PEI nanoparticles	IRF-4 mediated	HA-PEI–miR-125b NPs repolarized TAMs toward M1; in ovarian cancer model, combined with paclitaxel they significantly enhanced anti-tumor efficacy, reduced ascites and VEGF levels, without systemic toxicity [162]
miR-125b + miR-155 (dual)	HA-PEI/HA-PEG nanoparticles	IRF-4 and C/EBPβ	Strong macrophage activation under IFN-γ/LPS; robust M1 phenotype [163]
miR-155	sPEG/GLC nanocomplex	C/EBPβ; inflammatory transcriptional response	Effectively repolarized TAMs from an immunosuppressive M2 phenotype to an anti-tumor M1 state, evidenced by elevated IL-12, iNOS, and MHC II and reduced Msr2 and Arg1 expression. increase in activated T cells and NK cells within the tumor, ultimately producing strong tumor regression [164]
miR-155	Mannose-conjugated lipid-coated calcium phosphonate nanoparticles	Restores pro-inflammatory programming, activates SOCS1–NF-κB pathway	Repolarized TAMs to M1, increased CD8^+^ T-cell infiltration, reduced tumor growth and metastasis [165]
miR-125b	HA-PEI nanocarrier	IRF-4	Repolarization of lung TAMs to M1 phenotype [166].
DNA damage response 1 (Redd1)-siRNA	Outer membrane vesicles (OMVs) from gram-negative bacteria (siRNA@M-/PTX-CA-OMVs)	Redd1	Reduced Redd1 expression; altered M2-associated signaling; marked suppression of tumor progression and metastatic spread [167]
miR-127	CXCR4-targeting RNA-protein nanoplexes	JNK activation	Markedly inhibited both cancer and immune cell mobility, upregulated phosphorylated JNK kinase and M1 pro-inflammatory cytokines and suppressed tumor growth [168]
mRNA (IRF5 + IKKβ)	Nanoparticles consist of a PbAE-mRNA polyplex core coated with a layer of PGA-Di-mannose	IRF5 & IKKβ (M1-polarizing transcription factors)	IRF5/IKKβ NP delivery suppressed M2 genes (Serpinb2, Ccl11), boosted M1 markers (e.g., Ccl5), increased inflammatory monocytes and neutrophils, reduced suppressive macrophages, and enhanced lymphocyte infiltration, restoring anti-tumor immunity [108]
MGLL siRNA (siMGLL) and CB-2 siRNA (siCB-2)	Poly(disulfide amide) co-delivery nanosystem	MGLL, CB2 (metabolism + cannabinoid pathway)	The GSH-responsive NPs released siMGLL and siCB2 to silence both targets, suppress free fatty acid production, and repolarize TAMs toward an M1 phenotype. This dual metabolic and immune modulation boosted TNF-α/IL-12 secretion and produced strong tumor inhibition in xenograft and orthotopic PAC models [169].
siRNA—STAT3 or STAT6	Tyrosine-modified cationic polymeric NPs (PEI-based: LP10Y or P5Y; PPI-based: PPI-Y)	Knockdown of STAT3 or STAT6 (transcription factors driving M2 polarization)	STAT3 or STAT6 knockdown altered M1/M2 polarization markers; STAT6 knockdown via PPI-Y/siRNA notably repolarized M2 to M1-like phenotype and increased tumor-cell phagocytosis in co-culture, highlighting potential for TAM reprogramming [170]
mRNA encoding BisCCL2/5i	Lipid nanoparticle (LNP) platform	In situ expression of BisCCL2/5i to block CCL2/CCL5 signaling and repolarize TAMs toward an M1 phenotype	BisCCL2/5i mRNA–LNP combined with PD-1 blockade produced strong TAM repolarization, enhanced T-cell responses, and achieved long-term survival in both primary liver tumors and liver-metastatic models [171]
IKKβ siRNA	Diblock copolymer (N3-P[Lys(M2pep)-Lys]-PAsp- (DIP-co-BZA)) grafted with M2pep	Dual inhibition of STAT6 signaling and NF-κB activation to reverse M2 polarization and promote M1 phenotype	siRNA (IKKβ) + small-molecule STAT6 inhibitor co-delivery markedly suppressed M2 polarization, enhanced M1 activation, boosted antitumor immunity, and achieved strong tumor growth inhibition with minimal systemic immune toxicity [172]
STAT3 siRNA	Folic acid (FA)-conjugated liposomal nanobubbles	Activates IRF5 signaling, and disrupts JAK/STAT3 signaling, preventing macrophages from adopting an M2-like phenotype	US-responsive siRNA/Fe_3_O_4_ nanocarriers suppressed M2 polarization, promoted M1 macrophage activation, increased CD8^+^ T-cell infiltration, and significantly inhibited nonsmall cell lung cancer (NSCLC) tumor growth [173]

**Table 2:** Representative Nanoplatforms for Immunomodulation of TAMs and DCs in Cancer Therapy.

Nanoplatforms	Immunomodulators	Mechanisms	Targeting Tumor Model	Efficacy and Outcome
Albumin-based NP “Nano-PI”	IPI-549 (PI3Kγ inhibitor); paclitaxel (PTX), a chemotherapeutic drug). These 2 drugs were encapsulated by Nano-PI. α-PD1 (anti-programmed death) is injected separately	Passive TAM uptake via EPR; PI3Kγ inhibition reprograms TAMs; chemo-immunotherapy synergy	MMTV-PyMT transgenic mice, with either spontaneous or xenograft breast cancer models, as well as lung metastasis models	The combination of Nano-PI and α-PD1 led to more M2-M1 macrophage conversion, increased expression of CD4^+^ and CD8^+^ T cells, B cells, decreased regulatory T cells. Breast cancer models achieved long-term remission [47]
Phosphatase-like nanomaterial	Enzyme-mimetic nanomaterial	Autophagy induction in TAMs; metabolic reprogramming	B16 tumor-bearing C57BL/6 mice	Macrophage M2 to M1 shift; enhanced antitumor immunity and tumor suppression [113]
AEAA-PEG-PCL- based polymeric nanoparticles	Silibinin (anti-fibrotic compound) and IPI549 (PI3Kγ inhibitor)	Passive accumulation; TAM modulation via PI3Kγ blockade	Mouse 4T1 breast cancer xenograft model	Combination of treatments leads to higher therapeutic efficacy compared to treatments used individually; Significant decrease in regulatory T cells and myeloid-derived immune suppressor cells [117]
Core–shell Metal Organic Framework (MOF)-based nanomedicine (drug nanocore + MOF shell)	Nanocore made of IPI549 (PI3Kγ inhibitor), encapsulated by Toll-like 9 agonist (CpG)	Passive tumor accumulation; preferential macrophage uptake; intracellular release of IPI-549 and CpG synergistically reprograms TAMs from M2 to M1 phenotype; enhanced antigen presentation and cytokine secretion	Pulmonary metastasis melanoma model induced by IV injection of B16F10-luc cells in female C57BL/6 mice	Non-cytotoxic macrophage re-education; restored innate phagocytic activity; enhanced antigen presentation; increased CTL infiltration; synergistic tumor growth and metastasis inhibition when combined with anti-PD-L1 therapy [118]
Mannose-decorated porous hollow iron oxide NPs (PHNPs)	3-Methyladenine (3-MA), which is a PI3Kγ inhibitor	Mannose modifications served to increase TAM targeting Inhibited PI3K γ expression led to upregulation of the NF-κB p65 pathway	MDA-MB-231 tumor bearing Female Balb/c mice.	In vivo, TAM reprogramming led to increased CD8^+^ and CD4^+^ T cells, B cells, and T cells, while also decreasing Treg cells, providing evidence of the NP’s ability to modulate TME [119]
Lipid nanoparticle	CSF1R signaling axis+Anti-PD-L1 monoclonal antibody (mAB)	Targeting PD-L1-expressing macrophages, while inhibiting the PD-L1 checkpoint	Melanoma mouse model	Increase in M2-M1 conversion, as well as in the phagocytic index in vitro; increased CD8^+^ T-cell infiltration; superior anti-tumor efficacy at suboptimal doses; minimal systemic toxicity [127]
Polymersomes (PMs)-based delivery platform	Macrophage colony-stimulating factor 1 receptor inhibition (CSF1R inhibitor)	The blocking of CSF1/CSF1R pathways has been shown to lead to macrophage cell death, and lessening interaction with this pathway will lead to better anti-tumor results	MDA-MB-231, a breast cancer cell line	Effective M2-to-M1 repolarization of TAMs; increased pro-inflammatory cytokine secretion; enhanced CD8^+^ T-cell infiltration; significant tumor growth inhibition [128]
Intratumoral ATP-sensitive nanogel	BLZ-945 conjugated albumin AMD3100 (CSF-1R inhibitor); Paclitaxel (PTX; chemotherapeutic drug)	BLZ-945 mediates conversion of M2-M1 macrophages, AMD3100 decreases CXCR4 (a chemokine receptor) expression Both M2 and overexpression of chemokines in tumors serve to uphold the physical barriers that block chemotherapeutic medication from reaching the tumor	Mouse mammary carcinoma 4T1 cells and B16F10 tumor models	Nanogel displays the ability to deeply penetrate tumor membrane, allowing it to intratumorally block the CXCR4 receptors. This, along with the conversion of TAMs; improved tumor infiltration of cytotoxic T cells; synergistic chemo-immunotherapy effect allows for a decrease of tumor barriers and excites the anti-tumor immune response [129]
Cyclodextrin-based NPs	TLR7/9 resiquimod (R848), which is a receptor agonist.	M1-M2 polarization through agonization of Toll-like receptor (TLR) 7/8	MC38 mouse colon adenocarcinoma	Adamantane moiety improved resiquimod affinity to NP, leading to a reduction in the adverse side effects of R848 alone [138]
Cross-linked riboflavin-gelatin scaffold	TLR7/9 resiquimod (R848); this was solubilized with eugenol to make Resiquimod-GNE	M1-M2 polarization through agonization of Toll-like receptor (TLR) 7/8	M2-like murine macrophages (RAW 264.7 cells)	There was no toxicity to macrophages that received the treatment, meaning it can still perform its cellular functions after M2 to M1 conversion. The gelatin platform was stable in solution and was also biodegradable [140]
Mn-based nanoadjuvant (MPN/CpG)	Mn^2+^ and CpG combining into a nanoadjuvant (MPN/CpG)+EGCG	Mn^2+^ enhances CpG activity via STING–NF-κB pathway; repolarizes M2 to M1 TAMs; increases DC maturation and T-cell priming; improves lymph node accumulation	B16-OVA melanoma tumor-bearing mouse model	Reprograms TAMs to M1; MPN/CpG vaccination prevented tumor growth, boosted infiltration of CD8^+^ and CD4^+^ T cells and dendritic cells [141]
Acid-switchable NP (ability to release drug at different PHs)	IL-15 TGB-β inhibitor galunisertib	Promotion of NK cell activation via p-STAT5 and p-mTOR signaling (IL-15) Blocking TGB-β signaling enhanced cytotoxic receptor expression and granzyme B/perforin secretion	CT26 colorectal tumor model	NPs displayed pH-triggered aggregation, enhanced tumor retention, and sustained cytokine and drug release. NK cells and CD8^+^ T cells were activated, and M2-M1 polarization occurred, leading to a strong anti-tumor response [149].
Polyaniline-coated iron oxide NPs	Pani/y-Fe_2_O_3_ as an immunostimulatory agent	NPs increase labile metal pools and catalyze reactive oxidative species (ROS) generation inside of macrophages, upregulating TNF-α, IL-6, and iNOS, which all favor M1-like programs in macrophage	4T1 BALB/c model of metastatic breast cancer	In vivo, NP was able to reduce 4T1 tumor weight, increase M1 macrophage and NK cells, and suppress lung metastasis, all without traditional drug loading [177]
Superparamagnetic iron oxide NPs (SPION-CCPMs)	None (intrinsic nanoparticle effect)	Following phagocytosis of NPs, macrophages begin releasing reactive nitrogen species (RNS) and inflammatory cytokines, damaging nearby cancer cells	Murine lung adenocarcinoma (LUAD) model and Eml4-Alk lung cancer mouse model.	In vivo, NPs reshape immunosuppressive TME, increases CD8+ T cell infiltration and delays tumor growth. After first-line tyrosine kinase inhibitor therapy, SPION-CCPM significantly inhibits the regrowth of relapsing tumors [178]
Ferumoxytol (FDA-approved SPION)	Does not use traditional drug loading (intrinsic nanoparticle effect)	ROS burst from Fenton-type reactions elevated caspase-3 activation in cancer cells, indicating increased macrophage-mediated cytotoxicity in response to the NP	MMTV-PyMT-derived cancer cells were transplanted into female FVB/N mice for in vivo studies	Macrophages exposed to upregulated Th1-associated pro-inflammatory transcripts, reflecting a conversion to the M1 phenotype. NP reduced subcutaneous adenocarcinomas and lung cancer metastasis [179]
PEG-gold nanoparticles (PEG-AuNPs)	Does not use traditional drug loading (intrinsic nanoparticle effect)	PEG-AuNPs inhibit autophagic flux in TAMs by inducing lysosomal alkalization and membrane permeabilization, preventing the formation of autolysosomes	Hepa1-6 cells (mouse hepatoma cell line) injected into the right flank of male BALB/c mice with subcutaneous and xenografted liver cancer	In vitro, exposure to NPs caused M2-associated genes to be suppressed while pro-inflammatory pathways were activated. In the tumor-bearing mice models, autophagy inhibition by PEG-AuNPs enhanced infiltration of both CD3^+^CD4^+^ helper T cells and CD3^+^CD8^+^ cytotoxic T cells, resulting in suppression of tumor growth [182].
Mannose-functionalized graphene oxide (MGO)	LDN193189 (a multi-target small molecule inhibitor)	Mannose targets cancer stem cells (CSCs), which have receptors such as CD206; LDN193189 blocks BMP signaling pathway, binds to C-terminal domain of CD133	C57BL/6 mice injected with Hepa 1-6 cells (hepatocellular carcinoma cells)	The ability for the NP to target CSCs of the TME allows for greater anti-tumor effect. The medication itself, LDN193189, can alleviate T cell suppression, reverse immune evasion, and activate immune response. In vitro, NP has led to increased M2-M1 polarization [187]
Polymer-based composite antigen-capturing nanoparticles made of acid-ended poly(lactic-co-glycolic) acid (PLGA) and Polyethyleneimine (PEI)	Polyinosinic–polycytidylic acid (PIC), a toll-like receptor 3 (TLR3) agonist	The NP’s hydrophobic quality due to the PLGA, as well as the charged quality due to the PEI, allows for the capture of tumor proteins (antigens) such as PIC. These antigens could then be relayed to dendritic cells, such as CD103^+^ cDC1s, to facilitate increased antigen presentation	Multiple murine tumor models (MC38 colon carcinoma, melanoma, glioma)	NPs led to increased activation of CD103^+^ cDC1s; Combination of ACT-DC with immune checkpoint inhibitors, elimination of primary tumors in 50–100% of treated mice [204].
NP with polymer core and DOPC, cholesterol, Chol-HG, and DSPE-PEG in the lipid shell	Polyinosinic–polycytidylic acid (PPA)+3-(hydroxyolinyl) glycine (HG)	PPA is a TLR3 agonist, which increases M2-M1 polarization HG is a KDM3A inhibitor, which would downregulate CD47 and PD-L1 on tumor cells	Orthotopic triple-negative breast cancer (4T1) and pancreatic ductal adenocarcinoma models	Promotes M1 macrophage polarization and DC maturation; enhances CTL infiltration and activation; decreases regulatory T cells; significantly suppresses tumor progression and metastasis with minimal side effects [205]
Mesoporous silica nanoparticles (MSN), surface modified with NHS ester	Chlorin e6 (Ce6)+Plasmid DNA	pH-responsive tumor accumulation via PEG detachment; intracellular gene delivery encoding anti-PD-L1; laser-induced PDT triggers tumor cell death and boosts local release of PD-L1 antibody; enhances antigen release, dendritic cell maturation, and T-cell activation	B16F10-bearing mouse model	NP injection led to significant tumor suppression in both primary and bilateral tumor models; activated T cell maturation/infiltration [206]

## Data Availability

No new data were created or analyzed in this study. Data sharing is not applicable to this article.
